# Single Nucleotide Polymorphisms in *PPARD* Associated with Systemic Lupus Erythematosus in Chinese Populations

**DOI:** 10.1155/2020/7285747

**Published:** 2020-05-31

**Authors:** Yuan-yuan Qi, Ya-ling Zhai, Xin-ran Liu, Xiao-xue Zhang, Ya-fei Zhao, Xiang-hui Ning, Zhan-Zheng Zhao

**Affiliations:** ^1^Nephrology Hospital, The First Affiliated Hospital of Zhengzhou University, Henan 4500052, China; ^2^Institute of Nephrology, Zhengzhou University, Henan 4500052, China; ^3^Department of Urology, The First Affiliated Hospital of Zhengzhou University, Henan 4500052, China

## Abstract

**Background:**

Systemic lupus erythematosus (SLE) is a multifactorial autoimmune disease characterized by apoptotic clearance deficiency provoking autoimmune responses and leading to multiple organ damage. PPAR-*δ*, encoded by the *PPARD* gene, was induced in macrophages promoting the timely disposal of apoptotic cells. Biological studies had provided solid foundation of *PPARD* involvement in SLE; it is worthwhile to further explore the genetic contribution of *PPARD* to SLE.

**Methods:**

We performed a discovery-replication genetic association study. The discovery study was based on previous reported GWAS data. And the replication study was conducted in 1003 SLE patients and 815 healthy controls from Henan, Middle East of China. Further, we analyzed the eQTL effect to identify possible functional significance.

**Results:**

In the genetic association analysis, we observed significant association between the risk C allele of rs4713853 (*p* = 0.03, OR 1.167, 95% CI 1.015-1.341) and increased SLE susceptibility. Moreover, individuals with the risk C allele were associated with lower expression of *PPARD* and *DEF6*. Our clinical analysis showed that SLE patients with the risk C allele of rs4713853 were more likely to present a higher proportion of anti-Sm antibody presence (CC+CT vs. TT, 20.0% vs. 14.2%, *p* = 0.039) and higher level of Scr (median inter quarter range CC+CT vs. TT, 56 48-71 vs. 54 46-64 *μ*mol/L, *p* = 0.002).

**Conclusions:**

In conclusion, our study identified a novel association between *PPARD* rs4713853 and SLE susceptibility in Chinese populations. By integrating multiple layers of analysis, we suggested that *PPARD* might be a main candidate in the pathogenesis of SLE.

## 1. Introduction

Systemic lupus erythematosus (SLE) is a complex autoimmune disease with a strong genetic predisposition. In patients with SLE, the balance of generation and uptake of apoptotic cells by phagocytes is disrupted resulting in the persistent existence of apoptotic debris. Numerous studies have demonstrated that the dysfunction of apoptotic debris clearance might induce the loss of tolerance in autoimmunity leading to the overproduction of autoantibodies and multiple organ damage which are typical of SLE [1, 2]. Lupus nephritis (LN) is one of the most frequent and severe organ impairments of SLE. Decreased clearance of apoptotic cell debris with nucleoprotein autoantigens was associated with the presence of antinuclear or antidouble-stranded DNA antibodies contributing to lupus nephritis [3]. Double-stranded DNA antibodies were a predictor and could be detected in renal biopsy tissues in patients with lupus nephritis [4]. Thus, apoptotic clearance deficiency plays an important role in the initiation of autoimmune reactions in SLE [1].

Multiple molecules expressed on macrophages mediating the specific recognition and clearance of apoptotic debris were identified in SLE [1]. However, in genetic association studies, we only observed a robust genetic association between SLE and *ITGAM* which might impair leukocyte phagocytosis with the evidence from multiple populations indicating a possible genetic role for the dysfunction of apoptotic cell clearance [5–7]. Thus, the genetic basis for the clearance of apoptotic debris is still limited.

It is reported that the genetic deletion of *PPARD* (encode PPAR-*δ* which was expressed on macrophages) in mice presented with lupus manifestations [8]. As for genetic study, a significant association between PPAR-*γ* rs1805192 genotypes and decreased SLE risk was identified in a Chinese population [9]. But there was no association observed in a Korean population [10].

In the present study, we used the previous reported GWAS cohort from the Beijing population as the discovery cohort, and 1818 unrelated individuals were recruited from the Henan population as the replication cohort. We also analyzed the association between genetic phenotypes and clinical manifestations. Further bioinformatics analysis was also performed in order to find a possible functional role of the identified loci.

## 2. Materials and Methods

### 2.1. Case-Control Cohorts

The current genetic discovery-replication study was performed in 2 independent case-control cohorts. The case-control cohort used for the genetic discovery study was derived from a previously published GWAS cohort from Beijing, in the north of China. The recruitment procedure and criteria were described previously [11]. The replication cohort consists of 1003 SLE patients and 815 geographically matched unrelated healthy controls from Henan, in the middle east of China. All the patients fulfilled the criteria of the American College of Rheumatology for SLE. The study was approved by the Medical Ethics Committee of Zhengzhou University First Hospital (2019-KY-134).

### 2.2. SNP Selection and Genotyping

SNPs in the present study were included in *PPARD* which was located in chromosomes 6, 35, 310, 335-35, 395, 968 (GRCh37/hg19). 17 SNPs were included in the ImmunoChip array by the previous GWAS data, and the detailed genetic association results were provided in supplementary table 1 [11]. SNPs that survived from the Bonferroni correction can be further replicated in the replication cohort. We used Sequenom MassARRAY for genotyping in the replication cohort, and the genotyping yield was over 98% (detailed genotyping data for rs2267664 and rs4713853 were provided in supplementary table 2).

### 2.3. Bioinformatics and Expression Analysis

Since most of the candidate SNPs were intronic, we used the HaploReg database, which is a tool for exploring annotations of the noncoding genome at variants on haplotype blocks, to search for the possible functions (http://archive.broadinstitute.org/mammals/haploreg). The allele-dependent gene expression was queried using ArrayExpress (http://www.ebi.ac.uk/arrayexpress) and Ensemble (http://www.ensembl.org).

### 2.4. Statistical Analysis

Deviations from the Hardy-Weinberg equilibrium were calculated using a goodness-of-fit *χ*^2^ test (supplementary table 3). Allelic associations were assessed using a chi-square test to give the odds ratio with a 95% confidence interval. We performed Spearman's coefficient to calculate the correlation in the allele-dependent gene expression analysis. Quantitative variables with a normal distribution were expressed as the mean ± SD, and Student's *t*-test was performed. All statistical analyses were performed using SPSS 13.0 software. *p* < 0.05 was considered as statistically significant.

## 3. Result

### 3.1. *PPARD* Polymorphisms and SLE Risk in Chinese Population

In the genetic discovery stage, a total of 17 SNPs in the 86 kb *PPARD* region were successfully genotyped in the Chinese population by ImmunoChip. rs2267664 (*p* = 1.20∗10^−4^, OR 1.45, 95% CI 1.20-1.75) and rs4713853 (*p* = 4.15∗10^−4^, OR 1.39, 95% CI 1.16-1.68) were the top two signals included for further replication study (Table 1).

In the genetic replication stage, both rs2267664 (*p* = 0.05, OR 1.16, 95% CI 1.00-1.34) and rs4713853 (*p* = 0.03, OR 1.17, 95% CI 1.02-1.34) were successfully replicated in 1003 SLE patients and 815 healthy controls from a Chinese Henan population which was independent of the discovery Beijing cohort (Table 1). After we combined the results from the discovery-replication cohort, the genetic association of rs4713853 was further pronounced with *p*_meta_ = 1.77∗10^−4^.

### 3.2. Association Analysis of *PPARD* rs4713853 with Clinical Features

We further analyzed the association between rs4713853 and clinical manifestations of SLE patients. As shown in Table 2, SLE patients carrying the risk C allele (CC+CT) showed higher frequency of female patients and higher positive proportion of malar rash, discoid rash, photosensitivity, oral ulcers, nonerosive arthritis, pleuritis or pericarditis, leukopenia, thrombocytopenia, anti-dsDNA antibody, and anti-Sm antibody. We also observed a younger onset age, higher levels of serum creatinine (Scr), 24-hour total urine protein (24 h UTP), SLE disease activity index (SLEDAI), and lower levels of complements (C) 3 and C4 in rs4713853 CC+CT SLE patients.

More importantly, there was a significantly higher level of Scr (median interquarter range CC+CT vs. TT, 56 48-71 vs. 54 46-64 *μ*mol/L, *p* = 0.002), and higher proportions of anti-Sm antibody presence (CC+CT vs. TT, 20.0% vs. 14.2%, *p* = 0.039) were observed in SLE patients with CC+CT genotypes. Since the levels of serum creatinine are a continuous variable, we divided it by quartiles and performed the logistic regression analysis. The results showed that higher levels of Scr and the presence of an anti-Sm antibody were independently associated with CC+CT genotypes (the *p* value for the presence of the anti-Sm antibody was 0.042, and the *p* value for levels of Scr was 0.017).

### 3.3. Functional Annotation and eQTL Analysis

**Figure 1:**
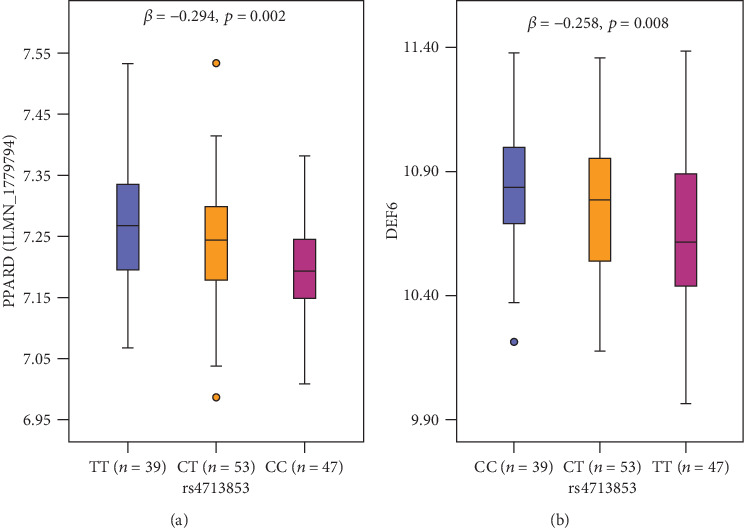
Expression QTL analysis in HapMap3 individuals. The box plot was a pooled analysis of the lymphoblast expression data available from 139 individuals including 43 CHB (Han Chinese in Beijing, China), 40 JPT (Japanese in Tokyo, Japan), and 55 YRI (Yoruba in Ibadan, Nigeria) populations.

As the associated variants mapped to intergenic and intronic regions, we first checked the functionality of the identified SNPs using HaploReg. rs4713853 was in high LD with regulatory variants, within the enhancer histone mark region in 18 tissues and within 14 altered motifs. As for eQTL analysis, we identified that rs4713853 was significantly associated with *UHRF1BP1*, *SNRPC*, *DEF6*, and ENSG00000065029.9 35262921 35263762 expression (Table 3). *PPARD* rs4713853 was also annotated to be expressed SNP by the lymphoblast expression data available from HapMap3 populations, as it showed that the rs4713853 CC (risk) genotype correlated with decreased *PPARD* (*p* = 0.002) and *DEF6* (*p* = 0.024) expression (Figure 1).

## 4. Discussion

Peroxisome proliferator-activated receptor delta (PPAR-*δ*) which is encoded by the *PPARD* gene belongs to the peroxisome proliferator-activated receptor (PPAR) family including PPAR-*α* (encoded by the *PPARA* gene), PPAR-*δ*, and PPAR-*γ* (encoded by the *PPARG* gene). The expression of PPAR-*δ* was induced in macrophages in response to apoptotic cells which promotes the timely disposal of apoptotic cells [8]. Genetic deletion of *PPARD* in mice showed lupus-like autoimmunity including higher levels of autoantibodies (in particular, the antinuclear antibody, ANA, and the double-stranded DNA, dsDNA) and renal impairment (increased urinary protein excretion, IgG deposition in glomeruli, and perivascular inflammation) [8]. Biological studies had provided solid foundation of *PPARD* involvement in SLE; it is worthwhile to further explore the genetic contribution of *PPARD* to SLE.

In the current study, we performed a genetic discovery-replication study to investigate into the impact of the variants on the *PPARD* gene and SLE risk in a Chinese population. Our data revealed that the potential functional rs4713853 was significantly associated with the susceptibility to SLE. Moreover, individuals with the risk C allele were associated with lower expression of *PPARD*. Accordingly, lower levels of *PPARD* mRNA expression were observed in PBMC and renal tubulointerstitium. Reduced expression of *PPARD* might lead to inefficient clearance of apoptotic cells provoking the breakdown of self-tolerance. The accumulation of apoptotic cells in the kidney might trigger inflammatory cytokine production promoting renal impairment. Our clinical analysis results supported this speculation and showed that patients with the risk C allele of rs4713853 were more likely to present an anti-Sm antibody and a higher level of Scr. In accordance with our findings, increased titers of autoantibodies and progressive renal impairment were confirmed in the *PPARD*^−/−^ mice. Taking together the findings from our genetic association analysis and previous in vivo model study [8], we hypothesized that patients carrying the risk C allele were associated with lower expression of *PPARD* which might cause the clearance dysfunction of apoptotic cells and the breakdown of self-tolerance, leading to increased production of autoantibody and renal impairment.

Our study also revealed that the risk C alleles were associated with lower expression of *DEF6*. DEF6, also known as IRF4 binding protein (IBP) [12] or SWAP-70-like adaptor of T cells (SLAT) [13], is a unique regulator of Rho GTPases which is required for maintaining T cell effector function, lymphocyte homeostasis, and the prevention of systemic autoimmunity [14]. Aging IBP-deficient (*IBP^trap/trap^*) female mice can develop a lupus-like syndrome spontaneously including high titers of autoantibody (ANA, anti-dsDNA, and anti-Sm antibodies) and glomerulonephritis [14]. Patients with *DEF6*-mutation presented with CTLA-4 haploinsufficiency and LRBA deficiency including T cell lymphopenia, low class-switched B cells, hepatosplenomegaly, autoimmune hemolytic anemia, and bowel inflammation [15–18]. Mutation in the *LRBA* gene is one of the causes of monogenic polyautoimmunity. Moreover, the frequencies of Th1, Th1-like Th17, and Th22 cells along with the expression of T-box transcription factor (TBET) and runt-related transcription factor 1 (RUNX1) are significantly increased in patients with LRBA [19]. Individuals with the risk C allele were associated with decreased expression of *DEF6* which might fail to regulate CTLA-4 availability and trafficking leading to overactivation of autoimmunity [16, 20, 21].

In conclusion, we verified the association between the *PPARD* gene rs4713853 risk C allele and increased SLE susceptibility, especially for immunological disorder and a higher level of Scr. Our study revealed a beneficial role of apoptotic cell clearance gene *PPARD* in the pathogenesis of SLE. Our findings would broaden the understanding of genetic contributions of the apoptotic cell clearance gene in SLE susceptibility.

## 5. Conclusions

Our study identified a novel association between *PPARD* rs4713853 and SLE susceptibility in Chinese populations. By integrating multiple layers of analysis, we suggested that *PPARD* might be a main candidate in the pathogenesis of SLE.

## Figures and Tables

**Table 1:** Association analysis of single nucleotide polymorphisms from *PPARD* gene with susceptibility to systemic lupus erythematosus in Chinese populations.

Chr.	Gene	SNP	Position (hg19)	Minor allele	Discovery stage (490/493)			Replication stage (1003/815)			Metaanalysis	
					MAF (case/control %)	*p* value	OR (95% CI)	MAF (case/control %)	*p* value	OR (95% CI)	*p* value	OR (95% CI)
6	*PPARD*	rs2267664	35312254	A	36.7/28.6	1.20∗10^−4^	1.45 (1.20-1.75)	30.3/27.4	0.05	1.16 (1.00-1.34)	2.19∗10^−4*a*^	1.24 (1.11-1.39)
6	*PPARD*	rs4713853	35327355	C	39.9/32.3	4.15∗10^−4^	1.39 (1.16-1.68)	34.9/31.5	0.03	1.17 (1.02-1.34)	1.77∗10^−4*a*^	1.24 (1.11-1.38)

^a^The *p* value threshold with Bonferroni correction was less than 0.00294118 when considering 17 variants.

**Table 2:** Stratification analysis for the association between *PPARD* rs4713853 polymorphism and SLE clinical manifestations.

Clinical manifestations		TT (*n* = 429)	CC+CT (*n* = 569)	*p* value
Gender (male, %)		24 (5.6%)	47 (8.3%)	0.105

Onset age (mean ± SD)		31.8 ± 13.0	30.9 ± 12.9	0.266

Malar rash (+, %)		102 (23.8%)	149 (26.2%)	0.385

Discoid rash (+, %)		2 (0.5%)	5 (0.9%)	0.697

Photosensitivity (+, %)		14 (3.3%)	28 (4.9%)	0.197

Oral ulcers (+, %)		29 (6.8%)	43 (7.6%)	0.630

Nonerosive arthritis (+, %)		118 (27.5%)	160 (28.1%)	0.830

Pleuritis or pericarditis (+, %)		28 (6.5%)	54 (9.5%)	0.131

Renal disorder	Serum creatinine (median, IQR)	54 (46-64)	56 (48-71)	0.002 (0.017^a^)
	24 h proteinuria (mean ± SD)	2.12 ± 2.80	2.71 ± 8.98	0.423

	Pathological classifications (I/II/III/IV/V, %)	0(0%)/4(5.3%)/21(28.0%)/23(30.7%)/27(36.0%)	1(0.8%)/14(11.2%)/18(14.4%)/49(39.2%)/43(34.4%)	0.086

Neurologic disorder (+, %)		18 (4.2%)	17 (3.0%)	0.304

Hematologic disorder	Hemolytic anemia (+, %)	9 (2.1%)	9 (1.6%)	0.544
	Leukopenia (+, %)	98 (23.5%)	149 (26.2%)	0.276
	Lymphopenia (+, %)	173 (41.7%)	230 (41.3%)	0.902
	Thrombocytopenia (+, %)	98 (23.6%)	145 (25.9%)	0.404

Immunologic disorder	Anti-dsDNA (+, %)	238 (61.8%)	331 (64.1%)	0.473
	Anti-Sm (+, %)	45 (14.2%)	87 (20.0%)	0.039 (0.042^b^)
	C3	0.74 ± 0.15	0.70 ± 0.34	0.082
	C4	0.15 ± 0.12	0.14 ± 0.13	0.271

SLEDAI (mean ± SD)		4.6 ± 4.2	4.7 ± 4.0	0.601

^a^ *p* value calculated using the quartile of serum creatinine under the logistic regression model. ^b^*p* value calculated under the logistic regression model.

**Table 3:** eQTL effect of rs4713853 in multiple tissues in HaploReg v4.1 database.

Study ID	PMID	Tissue	Correlated gene	*p* value
GTEx2015 v6	25954001	Esophagus gastroesophageal junction	*UHRF1BP1*	4.65∗10^−7^
GTEx2015 v6	25954001	Heart atrial appendage	*SNRPC*	7.20∗10^−6^
GTEx2015 v6	25954001	Muscle skeletal	*UHRF1BP1*	1.77∗10^−6^
GTEx2015 v6	25954001	Whole blood	*DEF6*	1.47∗10^−7^
Lappalainen2013	24037378	Lymphoblastoid EUR exon level	*ENSG00000065029.9 35262921 35263762*	1.59∗10^−6^

## Data Availability

The data that support the findings of this study are available from the corresponding author upon reasonable request.

## Abbreviations

SLE: Systemic lupus erythematosus
LN: Lupus nephritis
PPAR: Peroxisome proliferator-activated receptor
GWAS: Genome-wide association study
eQTL: Expression quantitative trait loci
SNP: Single nucleotide polymorphism
LD: Linkage disequilibrium
IBP: IRF4 binding protein
SLAT: SWAP-70-like adaptor of T cells.

## Supplementary Materials

Supplementary Materials Supplementary table 1: association results of SNPs in PPARD and SLE susceptibility. Supplementary table 2: genotyping data of rs4713853 and rs2267664 by Sequenom MassARRAY. Supplementary table 3. Hardy-Weinberg equilibrium for rs4713853 and rs2267664 in the replication cohort.
