# Supplementary material for: Single Nucleotide Polymorphisms in PPARD Associated with Systemic Lupus Erythematosus in Chinese Populations
Source: J Immunol Res. 2020 May 31;2020:7285747. doi: 10.1155/2020/7285747 (PMC7281840; doi:10.1155/2020/7285747)
Supplement: Supplementary Materials — Supplementary table 1: association results of SNPs in PPARD and SLE susceptibility. Supplementary table 2: genotyping data of rs4713853 and rs2267664 by Sequenom MassARRAY. Supplementary table 3. Hardy-Weinberg equilibrium for rs4713853 and rs2267664 in the replication cohort. [file 7285747.f1.docx]

**Single Nucleotide Polymorphisms in *PPARD* Associated with Systemic Lupus Erythematosus in Chinese Populations**

Yuan-yuan Qi^12^, Ya-ling Zhai^12^, Xin-ran Liu^12^, Xiao-xue Zhang^12^, Ya-fei Zhao^12^, Xiang-hui Ning^3^, Zhan-Zheng Zhao^12*^

AUTHORS’ INSTITUTION AND AFFILIATION

1.Nephrology Hospital, the First Affiliated Hospital of Zhengzhou University, Henan 4500052, China;

2. Institute of Nephrology, Zhengzhou University, Henan 4500052, China;

3. Department of Urology, the First Affiliated Hospital of Zhengzhou University, Henan 4500052, China.

CORRESPONDING AUTHOR

Dr. Zhan-zheng Zhao, MD & PhD;

Email: zhanzhengzhao@zzu.edu.cn

Nephrology Hospital, the First Affiliated Hospital of Zhengzhou University,

Institute of Nephrology, Zhengzhou University

No.1, Jianshe Road, Erqi District

Zhengzhou 4500052, P.R China

Supplementary table1. Association results of SNPs in *PPARD* and SLE susceptibility.

| SNP | Chr. | Pos (hg19) | Minor allele | MAF (Case/Control, %) | P-value | OR(95%CI) |
| --- | --- | --- | --- | --- | --- | --- |
| rs11571504 | 6 | 35310749 | A | 3.1/3.4 | 7.32*10^-1^ | 0.92(0.55-1.51) |
| rs2267664 | 6 | 35312254 | G | 36.7/28.6 | 1.20*10^-4α^ | 1.45(1.20-1.75) |
| rs73411747 | 6 | 35312668 | G | 2.4/2.7 | 6.87*10^-1^ | 0.89(0.51-1.56) |
| rs9658061 | 6 | 35313442 | C | 0.8/0.6 | 5.84*10^-1^ | 1.34(0.46-3.89) |
| rs9658068 | 6 | 35313976 | A | 7.4/7.4 | 9.72*10^-1^ | 0.99(0.71-1.39) |
| rs9658071 | 6 | 35315125 | G | 1.1/0.7 | 3.37*10^-1^ | 1.59(0.61-4.11) |
| imm_6_35426720 | 6 | 35318742 | T | 32.1/38.5 | 3.01*10^-3^ | 0.76(0.63-0.91) |
| rs7771323 | 6 | 35320448 | G | 0.6/0.5 | 7.55*10^-1^ | 1.21(0.37-3.97) |
| rs7744392 | 6 | 35322763 | G | 0.8/0.6 | 5.84*10^-1^ | 1.34(0.46-3.89) |
| rs7758272 | 6 | 35322975 | G | 0.8/0.6 | 5.84*10^-1^ | 1.34(0.46-3.89) |
| rs7746988 | 6 | 35324741 | C | 2.2/2.3 | 8.97*10^-1^ | 0.96(0.53-1.74) |
| rs73745198 | 6 | 35324894 | C | 0.3/0.7 | 2.08*10^-1^ | 0.43(0.11-1.67) |
| rs61333739 | 6 | 35326284 | G | 2.4/2.7 | 6.87*10^-1^ | 0.89(0.51-1.56) |
| rs4713853 | 6 | 35327355 | T | 39.9/32.3 | 4.15*10^-4α^ | 1.39(1.16-1.68) |
| rs9658077 | 6 | 35327361 | A | 0.3/0.7 | 2.08*10^-1^ | 0.43(0.11-1.67) |
| rs9658119 | 6 | 35365967 | C | 2.2/2.3 | 8.97*10^-1^ | 0.96(0.53-1.74) |

^α^: The p-value was considered significant after Bonferroni correction.

Supplementary table 2. Genotyping data of rs4713853 and rs2267664 by Sequenom Massarray.

| Sample ID | Group^a^ | rs4713853^b^ | rs2267664^c^ |
| --- | --- | --- | --- |
| HYJY2018C0001 | 1 | T | G |
| HYJY2018C0193 | 1 | T | G |
| HYJY2018C0009 | 1 | CT | AG |
| HYJY2018C0201 | 1 | T | G |
| HYJY2018C0017 | 1 | CT | AG |
| HYJY2018C0209 | 1 | CT | G |
| HYJY2018C0025 | 1 | CT | AG |
| HYJY2018C0217 | 1 | T | G |
| HYJY2018C0033 | 1 | CT | AG |
| HYJY2018C0225 | 1 | T | G |
| HYJY2018C0041 | 1 | CT | AG |
| HYJY2018C0233 | 1 | T | G |
| HYJY2018C0049 | 1 | T | G |
| HYJY2018C0241 | 1 | CT | AG |
| HYJY2018C0057 | 1 | T | G |
| HYJY2018C0249 | 1 | T | G |
| HYJY2018C0065 | 1 | CT | AG |
| HYJY2018C0257 | 1 | CT | AG |
| HYJY2018C0073 | 1 | T | G |
| HYJY2018C0265 | 1 | T | G |
| HYJY2018C0081 | 1 | CT | AG |
| HYJY2018C0273 | 1 | CT | AG |
| HYJY2018C0089 | 1 | CT | AG |
| HYJY2018C0281 | 1 | CT | AG |
| HYJY2018C0097 | 1 | C | A |
| HYJY2018C0289 | 1 | CT | AG |
| HYJY2018C0105 | 1 | T | G |
| HYJY2018C0297 | 1 | CT | AG |
| HYJY2018C0113 | 1 | T | G |
| HYJY2018C0305 | 1 | CT | AG |
| HYJY2018C0121 | 1 | CT | AG |
| HYJY2018C0313 | 1 | C | AG |
| HYJY2018C0129 | 1 | T | G |
| HYJY2018C0321 | 1 | T | G |
| HYJY2018C0137 | 1 | T | G |
| HYJY2018C0329 | 1 | T | G |
| HYJY2018C0145 | 1 | T | G |
| HYJY2018C0337 | 1 | T | G |
| HYJY2018C0153 | 1 | T | G |
| HYJY2018C0345 | 1 | CT | AG |
| HYJY2018C0161 | 1 | CT | AG |
| HYJY2018C0353 | 1 | CT | G |
| HYJY2018C0169 | 1 | C | AG |
| HYJY2018C0361 | 1 | N | G |
| HYJY2018C0177 | 1 | CT | AG |
| HYJY2018C0369 | 1 | C | A |
| HYJY2018C0185 | 1 | CT | AG |
| HYJY2018C0377 | 1 | CT | AG |
| HYJY2018C0002 | 1 | T | G |
| HYJY2018C0194 | 1 | CT | AG |
| HYJY2018C0010 | 1 | CT | AG |
| HYJY2018C0202 | 1 | T | G |
| HYJY2018C0018 | 1 | CT | AG |
| HYJY2018C0210 | 1 | CT | AG |
| HYJY2018C0026 | 1 | CT | AG |
| HYJY2018C0218 | 1 | CT | AG |
| HYJY2018C0034 | 1 | T | G |
| HYJY2018C0226 | 1 | CT | AG |
| HYJY2018C0042 | 1 | CT | AG |
| HYJY2018C0234 | 1 | CT | G |
| HYJY2018C0050 | 1 | CT | AG |
| HYJY2018C0242 | 1 | T | G |
| HYJY2018C0058 | 1 | T | G |
| HYJY2018C0250 | 1 | CT | AG |
| HYJY2018C0066 | 1 | T | G |
| HYJY2018C0258 | 1 | CT | AG |
| HYJY2018C0074 | 1 | T | G |
| HYJY2018C0266 | 1 | CT | G |
| HYJY2018C0082 | 1 | T | G |
| HYJY2018C0274 | 1 | T | G |
| HYJY2018C0090 | 1 | T | G |
| HYJY2018C0282 | 1 | CT | AG |
| HYJY2018C0098 | 1 | CT | AG |
| HYJY2018C0290 | 1 | T | G |
| HYJY2018C0106 | 1 | C | A |
| HYJY2018C0298 | 1 | CT | AG |
| HYJY2018C0114 | 1 | T | G |
| HYJY2018C0306 | 1 | T | G |
| HYJY2018C0122 | 1 | T | G |
| HYJY2018C0314 | 1 | T | G |
| HYJY2018C0130 | 1 | CT | AG |
| HYJY2018C0322 | 1 | T | G |
| HYJY2018C0138 | 1 | CT | AG |
| HYJY2018C0330 | 1 | T | G |
| HYJY2018C0146 | 1 | T | G |
| HYJY2018C0338 | 1 | T | G |
| HYJY2018C0154 | 1 | T | G |
| HYJY2018C0346 | 1 | CT | AG |
| HYJY2018C0162 | 1 | T | G |
| HYJY2018C0354 | 1 | CT | AG |
| HYJY2018C0170 | 1 | T | G |
| HYJY2018C0362 | 1 | T | G |
| HYJY2018C0178 | 1 | CT | AG |
| HYJY2018C0370 | 1 | CT | G |
| HYJY2018C0186 | 1 | CT | AG |
| HYJY2018C0378 | 1 | T | G |
| HYJY2018C0003 | 1 | T | N |
| HYJY2018C0195 | 1 | T | AG |
| HYJY2018C0011 | 1 | T | G |
| HYJY2018C0203 | 1 | T | G |
| HYJY2018C0019 | 1 | T | G |
| HYJY2018C0211 | 1 | T | G |
| HYJY2018C0027 | 1 | T | G |
| HYJY2018C0219 | 1 | T | G |
| HYJY2018C0035 | 1 | CT | AG |
| HYJY2018C0227 | 1 | T | G |
| HYJY2018C0043 | 1 | CT | AG |
| HYJY2018C0235 | 1 | CT | AG |
| HYJY2018C0051 | 1 | C | A |
| HYJY2018C0243 | 1 | CT | AG |
| HYJY2018C0059 | 1 | CT | AG |
| HYJY2018C0251 | 1 | T | G |
| HYJY2018C0067 | 1 | T | G |
| HYJY2018C0259 | 1 | CT | AG |
| HYJY2018C0075 | 1 | C | AG |
| HYJY2018C0267 | 1 | C | A |
| HYJY2018C0083 | 1 | CT | AG |
| HYJY2018C0275 | 1 | T | G |
| HYJY2018C0091 | 1 | T | G |
| HYJY2018C0283 | 1 | CT | AG |
| HYJY2018C0099 | 1 | T | G |
| HYJY2018C0291 | 1 | T | G |
| HYJY2018C0107 | 1 | CT | AG |
| HYJY2018C0299 | 1 | T | G |
| HYJY2018C0115 | 1 | CT | AG |
| HYJY2018C0307 | 1 | CT | AG |
| HYJY2018C0123 | 1 | T | G |
| HYJY2018C0315 | 1 | CT | AG |
| HYJY2018C0131 | 1 | T | G |
| HYJY2018C0323 | 1 | C | A |
| HYJY2018C0139 | 1 | T | G |
| HYJY2018C0331 | 1 | T | G |
| HYJY2018C0147 | 1 | CT | G |
| HYJY2018C0339 | 1 | T | G |
| HYJY2018C0155 | 1 | T | G |
| HYJY2018C0347 | 1 | T | G |
| HYJY2018C0163 | 1 | T | G |
| HYJY2018C0355 | 1 | CT | AG |
| HYJY2018C0171 | 1 | CT | AG |
| HYJY2018C0363 | 1 | CT | AG |
| HYJY2018C0179 | 1 | T | G |
| HYJY2018C0371 | 1 | CT | AG |
| HYJY2018C0187 | 1 | CT | AG |
| HYJY2018C0379 | 1 | C | AG |
| HYJY2018C0004 | 1 | T | G |
| HYJY2018C0196 | 1 | CT | AG |
| HYJY2018C0012 | 1 | T | G |
| HYJY2018C0204 | 1 | T | G |
| HYJY2018C0020 | 1 | T | G |
| HYJY2018C0212 | 1 | CT | AG |
| HYJY2018C0028 | 1 | CT | AG |
| HYJY2018C0220 | 1 | T | G |
| HYJY2018C0036 | 1 | CT | AG |
| HYJY2018C0228 | 1 | CT | AG |
| HYJY2018C0044 | 1 | T | G |
| HYJY2018C0236 | 1 | T | G |
| HYJY2018C0052 | 1 | T | G |
| HYJY2018C0244 | 1 | T | G |
| HYJY2018C0060 | 1 | CT | G |
| HYJY2018C0252 | 1 | T | G |
| HYJY2018C0068 | 1 | CT | AG |
| HYJY2018C0260 | 1 | T | G |
| HYJY2018C0076 | 1 | CT | AG |
| HYJY2018C0268 | 1 | T | G |
| HYJY2018C0084 | 1 | CT | AG |
| HYJY2018C0276 | 1 | C | A |
| HYJY2018C0092 | 1 | T | G |
| HYJY2018C0284 | 1 | C | A |
| HYJY2018C0100 | 1 | CT | N |
| HYJY2018C0292 | 1 | C | A |
| HYJY2018C0108 | 1 | T | G |
| HYJY2018C0300 | 1 | T | G |
| HYJY2018C0116 | 1 | CT | G |
| HYJY2018C0308 | 1 | T | G |
| HYJY2018C0124 | 1 | CT | AG |
| HYJY2018C0316 | 1 | CT | AG |
| HYJY2018C0132 | 1 | CT | AG |
| HYJY2018C0324 | 1 | C | A |
| HYJY2018C0140 | 1 | T | G |
| HYJY2018C0332 | 1 | CT | AG |
| HYJY2018C0148 | 1 | CT | AG |
| HYJY2018C0340 | 1 | T | G |
| HYJY2018C0156 | 1 | CT | AG |
| HYJY2018C0348 | 1 | T | G |
| HYJY2018C0164 | 1 | CT | G |
| HYJY2018C0356 | 1 | T | G |
| HYJY2018C0172 | 1 | CT | AG |
| HYJY2018C0364 | 1 | CT | AG |
| HYJY2018C0180 | 1 | T | G |
| HYJY2018C0372 | 1 | T | G |
| HYJY2018C0188 | 1 | T | G |
| HYJY2018C0380 | 1 | CT | AG |
| HYJY2018C0005 | 1 | CT | AG |
| HYJY2018C0197 | 1 | CT | AG |
| HYJY2018C0013 | 1 | CT | AG |
| HYJY2018C0205 | 1 | T | G |
| HYJY2018C0021 | 1 | CT | AG |
| HYJY2018C0213 | 1 | CT | AG |
| HYJY2018C0029 | 1 | T | G |
| HYJY2018C0221 | 1 | C | A |
| HYJY2018C0037 | 1 | CT | AG |
| HYJY2018C0229 | 1 | T | G |
| HYJY2018C0045 | 1 | T | G |
| HYJY2018C0237 | 1 | CT | AG |
| HYJY2018C0053 | 1 | T | G |
| HYJY2018C0245 | 1 | T | G |
| HYJY2018C0061 | 1 | T | G |
| HYJY2018C0253 | 1 | T | G |
| HYJY2018C0069 | 1 | CT | G |
| HYJY2018C0261 | 1 | CT | G |
| HYJY2018C0077 | 1 | T | G |
| HYJY2018C0269 | 1 | T | G |
| HYJY2018C0085 | 1 | CT | AG |
| HYJY2018C0277 | 1 | T | G |
| HYJY2018C0093 | 1 | T | G |
| HYJY2018C0285 | 1 | C | AG |
| HYJY2018C0101 | 1 | CT | AG |
| HYJY2018C0293 | 1 | CT | AG |
| HYJY2018C0109 | 1 | T | G |
| HYJY2018C0301 | 1 | T | G |
| HYJY2018C0117 | 1 | T | G |
| HYJY2018C0309 | 1 | T | G |
| HYJY2018C0125 | 1 | CT | AG |
| HYJY2018C0317 | 1 | T | G |
| HYJY2018C0133 | 1 | T | G |
| HYJY2018C0325 | 1 | CT | AG |
| HYJY2018C0141 | 1 | T | G |
| HYJY2018C0333 | 1 | CT | G |
| HYJY2018C0149 | 1 | CT | AG |
| HYJY2018C0341 | 1 | T | G |
| HYJY2018C0157 | 1 | T | G |
| HYJY2018C0349 | 1 | C | A |
| HYJY2018C0165 | 1 | CT | AG |
| HYJY2018C0357 | 1 | T | G |
| HYJY2018C0173 | 1 | CT | AG |
| HYJY2018C0365 | 1 | C | G |
| HYJY2018C0181 | 1 | CT | AG |
| HYJY2018C0373 | 1 | CT | AG |
| HYJY2018C0189 | 1 | T | G |
| HYJY2018C0381 | 1 | CT | AG |
| HYJY2018C0006 | 1 | CT | AG |
| HYJY2018C0198 | 1 | CT | AG |
| HYJY2018C0014 | 1 | C | A |
| HYJY2018C0206 | 1 | C | A |
| HYJY2018C0022 | 1 | CT | G |
| HYJY2018C0214 | 1 | CT | G |
| HYJY2018C0030 | 1 | T | G |
| HYJY2018C0222 | 1 | T | G |
| HYJY2018C0038 | 1 | T | G |
| HYJY2018C0230 | 1 | T | G |
| HYJY2018C0046 | 1 | C | A |
| HYJY2018C0238 | 1 | T | G |
| HYJY2018C0054 | 1 | CT | AG |
| HYJY2018C0246 | 1 | T | G |
| HYJY2018C0062 | 1 | CT | AG |
| HYJY2018C0254 | 1 | CT | G |
| HYJY2018C0070 | 1 | C | A |
| HYJY2018C0262 | 1 | CT | AG |
| HYJY2018C0078 | 1 | T | G |
| HYJY2018C0270 | 1 | T | G |
| HYJY2018C0086 | 1 | T | G |
| HYJY2018C0278 | 1 | T | G |
| HYJY2018C0094 | 1 | T | G |
| HYJY2018C0286 | 1 | T | G |
| HYJY2018C0102 | 1 | T | G |
| HYJY2018C0294 | 1 | CT | AG |
| HYJY2018C0110 | 1 | T | G |
| HYJY2018C0302 | 1 | CT | AG |
| HYJY2018C0118 | 1 | T | G |
| HYJY2018C0310 | 1 | T | G |
| HYJY2018C0126 | 1 | CT | AG |
| HYJY2018C0318 | 1 | T | G |
| HYJY2018C0134 | 1 | T | G |
| HYJY2018C0326 | 1 | T | G |
| HYJY2018C0142 | 1 | T | G |
| HYJY2018C0334 | 1 | C | A |
| HYJY2018C0150 | 1 | CT | AG |
| HYJY2018C0342 | 1 | CT | AG |
| HYJY2018C0158 | 1 | CT | AG |
| HYJY2018C0350 | 1 | T | G |
| HYJY2018C0166 | 1 | CT | AG |
| HYJY2018C0358 | 1 | T | G |
| HYJY2018C0174 | 1 | CT | AG |
| HYJY2018C0366 | 1 | T | G |
| HYJY2018C0182 | 1 | CT | AG |
| HYJY2018C0374 | 1 | T | G |
| HYJY2018C0190 | 1 | T | G |
| HYJY2018C0382 | 1 | CT | AG |
| HYJY2018C0007 | 1 | C | A |
| HYJY2018C0199 | 1 | CT | AG |
| HYJY2018C0015 | 1 | C | A |
| HYJY2018C0207 | 1 | CT | AG |
| HYJY2018C0023 | 1 | T | G |
| HYJY2018C0215 | 1 | T | G |
| HYJY2018C0031 | 1 | CT | G |
| HYJY2018C0223 | 1 | T | G |
| HYJY2018C0039 | 1 | T | G |
| HYJY2018C0231 | 1 | T | G |
| HYJY2018C0047 | 1 | T | G |
| HYJY2018C0239 | 1 | C | A |
| HYJY2018C0055 | 1 | C | A |
| HYJY2018C0247 | 1 | CT | AG |
| HYJY2018C0063 | 1 | T | G |
| HYJY2018C0255 | 1 | T | G |
| HYJY2018C0071 | 1 | T | G |
| HYJY2018C0263 | 1 | T | G |
| HYJY2018C0079 | 1 | CT | AG |
| HYJY2018C0271 | 1 | CT | AG |
| HYJY2018C0087 | 1 | T | G |
| HYJY2018C0279 | 1 | CT | AG |
| HYJY2018C0095 | 1 | CT | G |
| HYJY2018C0287 | 1 | CT | AG |
| HYJY2018C0103 | 1 | CT | AG |
| HYJY2018C0295 | 1 | CT | AG |
| HYJY2018C0111 | 1 | T | G |
| HYJY2018C0303 | 1 | CT | AG |
| HYJY2018C0119 | 1 | C | A |
| HYJY2018C0311 | 1 | T | G |
| HYJY2018C0127 | 1 | CT | G |
| HYJY2018C0319 | 1 | CT | AG |
| HYJY2018C0135 | 1 | T | G |
| HYJY2018C0327 | 1 | T | G |
| HYJY2018C0143 | 1 | T | G |
| HYJY2018C0335 | 1 | T | G |
| HYJY2018C0151 | 1 | T | G |
| HYJY2018C0343 | 1 | CT | AG |
| HYJY2018C0159 | 1 | T | G |
| HYJY2018C0351 | 1 | T | G |
| HYJY2018C0167 | 1 | T | G |
| HYJY2018C0359 | 1 | CT | AG |
| HYJY2018C0175 | 1 | T | G |
| HYJY2018C0367 | 1 | CT | AG |
| HYJY2018C0183 | 1 | CT | AG |
| HYJY2018C0375 | 1 | CT | AG |
| HYJY2018C0191 | 1 | T | G |
| HYJY2018C0383 | 1 | T | G |
| HYJY2018C0008 | 1 | T | G |
| HYJY2018C0200 | 1 | T | G |
| HYJY2018C0016 | 1 | T | G |
| HYJY2018C0208 | 1 | CT | AG |
| HYJY2018C0024 | 1 | CT | AG |
| HYJY2018C0216 | 1 | C | AG |
| HYJY2018C0032 | 1 | T | G |
| HYJY2018C0224 | 1 | T | G |
| HYJY2018C0040 | 1 | T | G |
| HYJY2018C0232 | 1 | T | G |
| HYJY2018C0048 | 1 | T | G |
| HYJY2018C0240 | 1 | CT | AG |
| HYJY2018C0056 | 1 | CT | G |
| HYJY2018C0248 | 1 | T | G |
| HYJY2018C0064 | 1 | CT | AG |
| HYJY2018C0256 | 1 | CT | AG |
| HYJY2018C0072 | 1 | T | G |
| HYJY2018C0264 | 1 | CT | AG |
| HYJY2018C0080 | 1 | T | G |
| HYJY2018C0272 | 1 | CT | AG |
| HYJY2018C0088 | 1 | T | G |
| HYJY2018C0280 | 1 | CT | AG |
| HYJY2018C0096 | 1 | CT | AG |
| HYJY2018C0288 | 1 | T | G |
| HYJY2018C0104 | 1 | T | G |
| HYJY2018C0296 | 1 | CT | AG |
| HYJY2018C0112 | 1 | CT | AG |
| HYJY2018C0304 | 1 | CT | AG |
| HYJY2018C0120 | 1 | T | G |
| HYJY2018C0312 | 1 | CT | G |
| HYJY2018C0128 | 1 | N | N |
| HYJY2018C0320 | 1 | T | G |
| HYJY2018C0136 | 1 | T | G |
| HYJY2018C0328 | 1 | T | G |
| HYJY2018C0144 | 1 | T | G |
| HYJY2018C0336 | 1 | CT | N |
| HYJY2018C0152 | 1 | CT | AG |
| HYJY2018C0344 | 1 | T | G |
| HYJY2018C0160 | 1 | C | AG |
| HYJY2018C0352 | 1 | C | AG |
| HYJY2018C0168 | 1 | CT | AG |
| HYJY2018C0360 | 1 | T | G |
| HYJY2018C0176 | 1 | CT | AG |
| HYJY2018C0368 | 1 | T | G |
| HYJY2018C0184 | 1 | CT | G |
| HYJY2018C0376 | 1 | T | G |
| HYJY2018C0192 | 1 | CT | AG |
| HYJY2018C0384 | 1 | CT | AG |
| HYJY2018C0385 | 1 | CT | AG |
| HYJY2018C0577 | 1 | CT | G |
| HYJY2018C0393 | 1 | T | G |
| HYJY2018C0585 | 1 | T | G |
| HYJY2018C0401 | 1 | CT | AG |
| HYJY2018C0593 | 1 | CT | AG |
| HYJY2018C0409 | 1 | CT | AG |
| HYJY2018C0601 | 1 | CT | AG |
| HYJY2018C0417 | 1 | CT | AG |
| HYJY2018C0609 | 1 | CT | AG |
| HYJY2018C0425 | 1 | N | N |
| HYJY2018C0617 | 1 | T | G |
| HYJY2018C0433 | 1 | CT | G |
| HYJY2018C0625 | 1 | CT | AG |
| HYJY2018C0441 | 1 | CT | AG |
| HYJY2018C0633 | 1 | CT | AG |
| HYJY2018C0449 | 1 | CT | AG |
| HYJY2018C0641 | 1 | T | G |
| HYJY2018C0457 | 1 | T | G |
| HYJY2018C0649 | 1 | CT | AG |
| HYJY2018C0465 | 1 | CT | AG |
| HYJY2018C0657 | 1 | T | G |
| HYJY2018C0473 | 1 | CT | AG |
| HYJY2018C0665 | 1 | CT | AG |
| HYJY2018C0481 | 1 | T | G |
| HYJY2018C0673 | 1 | C | A |
| HYJY2018C0489 | 1 | CT | AG |
| HYJY2018C0681 | 1 | T | G |
| HYJY2018C0497 | 1 | CT | AG |
| HYJY2018C0689 | 1 | CT | AG |
| HYJY2018C0505 | 1 | T | G |
| HYJY2018C0697 | 1 | T | G |
| HYJY2018C0513 | 1 | T | G |
| HYJY2018C0705 | 1 | T | G |
| HYJY2018C0521 | 1 | CT | AG |
| HYJY2018C0713 | 1 | T | G |
| HYJY2018C0529 | 1 | CT | AG |
| HYJY2018C0721 | 1 | T | G |
| HYJY2018C0537 | 1 | C | A |
| HYJY2018C0729 | 1 | C | AG |
| HYJY2018C0545 | 1 | T | G |
| HYJY2018C0737 | 1 | CT | AG |
| HYJY2018C0553 | 1 | T | G |
| HYJY2018C0745 | 1 | T | G |
| HYJY2018C0561 | 1 | CT | AG |
| HYJY2018C0753 | 1 | CT | AG |
| HYJY2018C0569 | 1 | CT | AG |
| HYJY2018C0761 | 1 | CT | AG |
| HYJY2018C0386 | 1 | T | G |
| HYJY2018C0578 | 1 | T | G |
| HYJY2018C0394 | 1 | T | G |
| HYJY2018C0586 | 1 | C | A |
| HYJY2018C0402 | 1 | CT | AG |
| HYJY2018C0594 | 1 | CT | AG |
| HYJY2018C0410 | 1 | CT | AG |
| HYJY2018C0602 | 1 | CT | AG |
| HYJY2018C0418 | 1 | CT | AG |
| HYJY2018C0610 | 1 | CT | AG |
| HYJY2018C0426 | 1 | CT | AG |
| HYJY2018C0618 | 1 | T | G |
| HYJY2018C0434 | 1 | CT | AG |
| HYJY2018C0626 | 1 | CT | AG |
| HYJY2018C0442 | 1 | CT | AG |
| HYJY2018C0634 | 1 | CT | AG |
| HYJY2018C0450 | 1 | C | A |
| HYJY2018C0642 | 1 | C | AG |
| HYJY2018C0458 | 1 | CT | G |
| HYJY2018C0650 | 1 | CT | AG |
| HYJY2018C0466 | 1 | CT | AG |
| HYJY2018C0658 | 1 | CT | N |
| HYJY2018C0474 | 1 | T | G |
| HYJY2018C0666 | 1 | T | G |
| HYJY2018C0482 | 1 | T | G |
| HYJY2018C0674 | 1 | CT | AG |
| HYJY2018C0490 | 1 | CT | AG |
| HYJY2018C0682 | 1 | C | AG |
| HYJY2018C0498 | 1 | T | G |
| HYJY2018C0690 | 1 | C | A |
| HYJY2018C0506 | 1 | T | G |
| HYJY2018C0698 | 1 | CT | AG |
| HYJY2018C0514 | 1 | CT | AG |
| HYJY2018C0706 | 1 | T | G |
| HYJY2018C0522 | 1 | T | G |
| HYJY2018C0714 | 1 | CT | AG |
| HYJY2018C0530 | 1 | CT | AG |
| HYJY2018C0722 | 1 | T | G |
| HYJY2018C0538 | 1 | CT | AG |
| HYJY2018C0730 | 1 | T | G |
| HYJY2018C0546 | 1 | CT | AG |
| HYJY2018C0738 | 1 | CT | AG |
| HYJY2018C0554 | 1 | CT | AG |
| HYJY2018C0746 | 1 | C | A |
| HYJY2018C0562 | 1 | T | N |
| HYJY2018C0754 | 1 | T | G |
| HYJY2018C0570 | 1 | T | G |
| HYJY2018C0762 | 1 | CT | AG |
| HYJY2018C0387 | 1 | T | G |
| HYJY2018C0579 | 1 | T | G |
| HYJY2018C0395 | 1 | T | G |
| HYJY2018C0587 | 1 | T | G |
| HYJY2018C0403 | 1 | CT | AG |
| HYJY2018C0595 | 1 | T | G |
| HYJY2018C0411 | 1 | C | A |
| HYJY2018C0603 | 1 | T | G |
| HYJY2018C0419 | 1 | CT | AG |
| HYJY2018C0611 | 1 | CT | AG |
| HYJY2018C0427 | 1 | T | G |
| HYJY2018C0619 | 1 | T | G |
| HYJY2018C0435 | 1 | T | G |
| HYJY2018C0627 | 1 | T | G |
| HYJY2018C0443 | 1 | T | G |
| HYJY2018C0635 | 1 | T | G |
| HYJY2018C0451 | 1 | T | G |
| HYJY2018C0643 | 1 | C | A |
| HYJY2018C0459 | 1 | CT | AG |
| HYJY2018C0651 | 1 | T | G |
| HYJY2018C0467 | 1 | T | G |
| HYJY2018C0659 | 1 | C | A |
| HYJY2018C0475 | 1 | T | G |
| HYJY2018C0667 | 1 | T | G |
| HYJY2018C0483 | 1 | T | G |
| HYJY2018C0675 | 1 | T | G |
| HYJY2018C0491 | 1 | CT | AG |
| HYJY2018C0683 | 1 | T | G |
| HYJY2018C0499 | 1 | C | G |
| HYJY2018C0691 | 1 | CT | G |
| HYJY2018C0507 | 1 | CT | AG |
| HYJY2018C0699 | 1 | C | A |
| HYJY2018C0515 | 1 | T | G |
| HYJY2018C0707 | 1 | C | A |
| HYJY2018C0523 | 1 | C | A |
| HYJY2018C0715 | 1 | CT | AG |
| HYJY2018C0531 | 1 | T | G |
| HYJY2018C0723 | 1 | CT | AG |
| HYJY2018C0539 | 1 | T | G |
| HYJY2018C0731 | 1 | T | G |
| HYJY2018C0547 | 1 | T | G |
| HYJY2018C0739 | 1 | CT | AG |
| HYJY2018C0555 | 1 | C | A |
| HYJY2018C0747 | 1 | CT | AG |
| HYJY2018C0563 | 1 | T | G |
| HYJY2018C0755 | 1 | CT | AG |
| HYJY2018C0571 | 1 | T | G |
| HYJY2018C0763 | 1 | CT | G |
| HYJY2018C0388 | 1 | CT | AG |
| HYJY2018C0580 | 1 | T | G |
| HYJY2018C0396 | 1 | C | AG |
| HYJY2018C0588 | 1 | T | G |
| HYJY2018C0404 | 1 | CT | AG |
| HYJY2018C0596 | 1 | CT | AG |
| HYJY2018C0412 | 1 | CT | AG |
| HYJY2018C0604 | 1 | C | A |
| HYJY2018C0420 | 1 | T | G |
| HYJY2018C0612 | 1 | T | G |
| HYJY2018C0428 | 1 | CT | AG |
| HYJY2018C0620 | 1 | T | G |
| HYJY2018C0436 | 1 | CT | AG |
| HYJY2018C0628 | 1 | C | A |
| HYJY2018C0444 | 1 | CT | N |
| HYJY2018C0636 | 1 | C | A |
| HYJY2018C0452 | 1 | T | G |
| HYJY2018C0644 | 1 | T | G |
| HYJY2018C0460 | 1 | T | G |
| HYJY2018C0652 | 1 | T | G |
| HYJY2018C0468 | 1 | CT | AG |
| HYJY2018C0660 | 1 | C | A |
| HYJY2018C0476 | 1 | T | G |
| HYJY2018C0668 | 1 | T | G |
| HYJY2018C0484 | 1 | CT | AG |
| HYJY2018C0676 | 1 | T | G |
| HYJY2018C0492 | 1 | T | G |
| HYJY2018C0684 | 1 | T | G |
| HYJY2018C0500 | 1 | CT | AG |
| HYJY2018C0692 | 1 | CT | A |
| HYJY2018C0508 | 1 | CT | G |
| HYJY2018C0700 | 1 | C | AG |
| HYJY2018C0516 | 1 | T | G |
| HYJY2018C0708 | 1 | T | G |
| HYJY2018C0524 | 1 | CT | AG |
| HYJY2018C0716 | 1 | T | G |
| HYJY2018C0532 | 1 | CT | AG |
| HYJY2018C0724 | 1 | C | A |
| HYJY2018C0540 | 1 | C | AG |
| HYJY2018C0732 | 1 | N | N |
| HYJY2018C0548 | 1 | T | G |
| HYJY2018C0740 | 1 | T | G |
| HYJY2018C0556 | 1 | CT | AG |
| HYJY2018C0748 | 1 | T | G |
| HYJY2018C0564 | 1 | CT | AG |
| HYJY2018C0756 | 1 | T | G |
| HYJY2018C0572 | 1 | CT | AG |
| HYJY2018C0764 | 1 | T | G |
| HYJY2018C0389 | 1 | CT | AG |
| HYJY2018C0581 | 1 | CT | AG |
| HYJY2018C0397 | 1 | CT | AG |
| HYJY2018C0589 | 1 | C | A |
| HYJY2018C0405 | 1 | CT | AG |
| HYJY2018C0597 | 1 | CT | AG |
| HYJY2018C0413 | 1 | CT | AG |
| HYJY2018C0605 | 1 | T | G |
| HYJY2018C0421 | 1 | CT | AG |
| HYJY2018C0613 | 1 | CT | AG |
| HYJY2018C0429 | 1 | CT | G |
| HYJY2018C0621 | 1 | CT | AG |
| HYJY2018C0437 | 1 | T | G |
| HYJY2018C0629 | 1 | CT | G |
| HYJY2018C0445 | 1 | T | G |
| HYJY2018C0637 | 1 | CT | AG |
| HYJY2018C0453 | 1 | T | G |
| HYJY2018C0645 | 1 | T | G |
| HYJY2018C0461 | 1 | CT | AG |
| HYJY2018C0653 | 1 | T | G |
| HYJY2018C0469 | 1 | CT | G |
| HYJY2018C0661 | 1 | CT | AG |
| HYJY2018C0477 | 1 | T | G |
| HYJY2018C0669 | 1 | T | G |
| HYJY2018C0485 | 1 | CT | AG |
| HYJY2018C0677 | 1 | T | G |
| HYJY2018C0493 | 1 | CT | AG |
| HYJY2018C0685 | 1 | CT | AG |
| HYJY2018C0501 | 1 | CT | AG |
| HYJY2018C0693 | 1 | CT | AG |
| HYJY2018C0509 | 1 | CT | N |
| HYJY2018C0701 | 1 | T | G |
| HYJY2018C0517 | 1 | T | G |
| HYJY2018C0709 | 1 | CT | AG |
| HYJY2018C0525 | 1 | CT | AG |
| HYJY2018C0717 | 1 | T | G |
| HYJY2018C0533 | 1 | CT | AG |
| HYJY2018C0725 | 1 | CT | G |
| HYJY2018C0541 | 1 | CT | AG |
| HYJY2018C0733 | 1 | CT | AG |
| HYJY2018C0549 | 1 | CT | G |
| HYJY2018C0741 | 1 | T | G |
| HYJY2018C0557 | 1 | T | G |
| HYJY2018C0749 | 1 | CT | AG |
| HYJY2018C0565 | 1 | T | G |
| HYJY2018C0757 | 1 | T | G |
| HYJY2018C0573 | 1 | T | G |
| HYJY2018C0765 | 1 | T | G |
| HYJY2018C0390 | 1 | CT | AG |
| HYJY2018C0582 | 1 | T | G |
| HYJY2018C0398 | 1 | C | A |
| HYJY2018C0590 | 1 | T | G |
| HYJY2018C0406 | 1 | T | G |
| HYJY2018C0598 | 1 | T | G |
| HYJY2018C0414 | 1 | CT | AG |
| HYJY2018C0606 | 1 | CT | AG |
| HYJY2018C0422 | 1 | C | A |
| HYJY2018C0614 | 1 | CT | AG |
| HYJY2018C0430 | 1 | T | G |
| HYJY2018C0622 | 1 | CT | AG |
| HYJY2018C0438 | 1 | CT | AG |
| HYJY2018C0630 | 1 | CT | AG |
| HYJY2018C0446 | 1 | CT | AG |
| HYJY2018C0638 | 1 | CT | AG |
| HYJY2018C0454 | 1 | T | G |
| HYJY2018C0646 | 1 | T | G |
| HYJY2018C0462 | 1 | C | A |
| HYJY2018C0654 | 1 | CT | AG |
| HYJY2018C0470 | 1 | C | G |
| HYJY2018C0662 | 1 | T | G |
| HYJY2018C0478 | 1 | CT | AG |
| HYJY2018C0670 | 1 | CT | AG |
| HYJY2018C0486 | 1 | T | G |
| HYJY2018C0678 | 1 | T | G |
| HYJY2018C0494 | 1 | CT | AG |
| HYJY2018C0686 | 1 | T | G |
| HYJY2018C0502 | 1 | CT | AG |
| HYJY2018C0694 | 1 | T | G |
| HYJY2018C0510 | 1 | CT | AG |
| HYJY2018C0702 | 1 | T | G |
| HYJY2018C0518 | 1 | CT | AG |
| HYJY2018C0710 | 1 | CT | AG |
| HYJY2018C0526 | 1 | T | G |
| HYJY2018C0718 | 1 | T | G |
| HYJY2018C0534 | 1 | CT | AG |
| HYJY2018C0726 | 1 | C | A |
| HYJY2018C0542 | 1 | CT | AG |
| HYJY2018C0734 | 1 | CT | AG |
| HYJY2018C0550 | 1 | T | G |
| HYJY2018C0742 | 1 | CT | AG |
| HYJY2018C0558 | 1 | CT | G |
| HYJY2018C0750 | 1 | CT | AG |
| HYJY2018C0566 | 1 | CT | G |
| HYJY2018C0758 | 1 | T | G |
| HYJY2018C0574 | 1 | CT | G |
| HYJY2018C0766 | 1 | T | G |
| HYJY2018C0391 | 1 | CT | AG |
| HYJY2018C0583 | 1 | T | G |
| HYJY2018C0399 | 1 | CT | AG |
| HYJY2018C0591 | 1 | T | G |
| HYJY2018C0407 | 1 | T | G |
| HYJY2018C0599 | 1 | T | G |
| HYJY2018C0415 | 1 | T | G |
| HYJY2018C0607 | 1 | CT | AG |
| HYJY2018C0423 | 1 | CT | AG |
| HYJY2018C0615 | 1 | C | A |
| HYJY2018C0431 | 1 | T | G |
| HYJY2018C0623 | 1 | T | G |
| HYJY2018C0439 | 1 | CT | G |
| HYJY2018C0631 | 1 | T | G |
| HYJY2018C0447 | 1 | CT | AG |
| HYJY2018C0639 | 1 | CT | AG |
| HYJY2018C0455 | 1 | T | G |
| HYJY2018C0647 | 1 | CT | AG |
| HYJY2018C0463 | 1 | CT | AG |
| HYJY2018C0655 | 1 | CT | AG |
| HYJY2018C0471 | 1 | CT | AG |
| HYJY2018C0663 | 1 | T | G |
| HYJY2018C0479 | 1 | T | G |
| HYJY2018C0671 | 1 | CT | AG |
| HYJY2018C0487 | 1 | CT | AG |
| HYJY2018C0679 | 1 | T | G |
| HYJY2018C0495 | 1 | CT | G |
| HYJY2018C0687 | 1 | C | AG |
| HYJY2018C0503 | 1 | T | G |
| HYJY2018C0695 | 1 | T | G |
| HYJY2018C0511 | 1 | CT | AG |
| HYJY2018C0703 | 1 | CT | AG |
| HYJY2018C0519 | 1 | CT | AG |
| HYJY2018C0711 | 1 | CT | G |
| HYJY2018C0527 | 1 | CT | G |
| HYJY2018C0719 | 1 | T | G |
| HYJY2018C0535 | 1 | T | G |
| HYJY2018C0727 | 1 | T | G |
| HYJY2018C0543 | 1 | T | G |
| HYJY2018C0735 | 1 | T | G |
| HYJY2018C0551 | 1 | T | G |
| HYJY2018C0743 | 1 | CT | AG |
| HYJY2018C0559 | 1 | T | G |
| HYJY2018C0751 | 1 | CT | AG |
| HYJY2018C0567 | 1 | CT | AG |
| HYJY2018C0759 | 1 | CT | AG |
| HYJY2018C0575 | 1 | T | G |
| HYJY2018C0767 | 1 | T | G |
| HYJY2018C0392 | 1 | T | G |
| HYJY2018C0584 | 1 | CT | AG |
| HYJY2018C0400 | 1 | T | G |
| HYJY2018C0592 | 1 | T | G |
| HYJY2018C0408 | 1 | CT | AG |
| HYJY2018C0600 | 1 | T | G |
| HYJY2018C0416 | 1 | C | A |
| HYJY2018C0608 | 1 | T | G |
| HYJY2018C0424 | 1 | CT | AG |
| HYJY2018C0616 | 1 | CT | AG |
| HYJY2018C0432 | 1 | T | G |
| HYJY2018C0624 | 1 | T | G |
| HYJY2018C0440 | 1 | T | G |
| HYJY2018C0632 | 1 | T | G |
| HYJY2018C0448 | 1 | T | G |
| HYJY2018C0640 | 1 | C | A |
| HYJY2018C0456 | 1 | T | G |
| HYJY2018C0648 | 1 | T | G |
| HYJY2018C0464 | 1 | CT | AG |
| HYJY2018C0656 | 1 | CT | AG |
| HYJY2018C0472 | 1 | T | G |
| HYJY2018C0664 | 1 | CT | AG |
| HYJY2018C0480 | 1 | T | G |
| HYJY2018C0672 | 1 | T | G |
| HYJY2018C0488 | 1 | CT | AG |
| HYJY2018C0680 | 1 | T | G |
| HYJY2018C0496 | 1 | T | G |
| HYJY2018C0688 | 1 | T | G |
| HYJY2018C0504 | 1 | C | A |
| HYJY2018C0696 | 1 | CT | G |
| HYJY2018C0512 | 1 | CT | N |
| HYJY2018C0704 | 1 | CT | G |
| HYJY2018C0520 | 1 | CT | AG |
| HYJY2018C0712 | 1 | T | G |
| HYJY2018C0528 | 1 | T | G |
| HYJY2018C0720 | 1 | T | G |
| HYJY2018C0536 | 1 | CT | AG |
| HYJY2018C0728 | 1 | CT | AG |
| HYJY2018C0544 | 1 | CT | G |
| HYJY2018C0736 | 1 | C | A |
| HYJY2018C0552 | 1 | T | G |
| HYJY2018C0744 | 1 | CT | AG |
| HYJY2018C0560 | 1 | T | G |
| HYJY2018C0752 | 1 | CT | AG |
| HYJY2018C0568 | 1 | C | A |
| HYJY2018C0760 | 1 | T | G |
| HYJY2018C0576 | 1 | T | G |
| HYJY2018C0768 | 1 | T | G |
| HYJY2018C0769 | 1 | T | G |
| HYJY2018C0777 | 1 | T | G |
| HYJY2018C0785 | 1 | CT | AG |
| HYJY2018C0793 | 1 | T | G |
| HYJY2018C0801 | 1 | CT | AG |
| HYJY2018C0809 | 1 | C | A |
| HYJY2018C0770 | 1 | CT | AG |
| HYJY2018C0778 | 1 | T | G |
| HYJY2018C0786 | 1 | T | G |
| HYJY2018C0794 | 1 | CT | AG |
| HYJY2018C0802 | 1 | CT | G |
| HYJY2018C0810 | 1 | CT | G |
| HYJY2018C0771 | 1 | CT | AG |
| HYJY2018C0779 | 1 | T | G |
| HYJY2018C0787 | 1 | CT | AG |
| HYJY2018C0795 | 1 | C | A |
| HYJY2018C0803 | 1 | CT | AG |
| HYJY2018C0811 | 1 | CT | AG |
| HYJY2018C0772 | 1 | T | G |
| HYJY2018C0780 | 1 | T | G |
| HYJY2018C0788 | 1 | CT | AG |
| HYJY2018C0796 | 1 | T | G |
| HYJY2018C0804 | 1 | CT | AG |
| HYJY2018C0812 | 1 | T | G |
| HYJY2018C0773 | 1 | CT | G |
| HYJY2018C0781 | 1 | T | G |
| HYJY2018C0789 | 1 | CT | G |
| HYJY2018C0797 | 1 | CT | AG |
| HYJY2018C0805 | 1 | CT | AG |
| HYJY2018C0813 | 1 | T | G |
| HYJY2018C0774 | 1 | C | A |
| HYJY2018C0782 | 1 | T | G |
| HYJY2018C0790 | 1 | CT | AG |
| HYJY2018C0798 | 1 | CT | AG |
| HYJY2018C0806 | 1 | CT | AG |
| HYJY2018C0814 | 1 | CT | AG |
| HYJY2018C0775 | 1 | T | G |
| HYJY2018C0783 | 1 | T | G |
| HYJY2018C0791 | 1 | CT | AG |
| HYJY2018C0799 | 1 | CT | AG |
| HYJY2018C0807 | 1 | T | G |
| HYJY2018C0815 | 1 | T | G |
| HYJY2018C0776 | 1 | T | G |
| HYJY2018C0784 | 1 | T | G |
| HYJY2018C0792 | 1 | C | A |
| HYJY2018C0800 | 1 | T | G |
| HYJY2018C0808 | 1 | C | A |
| 20180001 | 2 | T | G |
| 20180002 | 2 | CT | AG |
| 20180003 | 2 | CT | AG |
| 20180004 | 2 | T | G |
| 20180006 | 2 | T | G |
| 20180008 | 2 | T | G |
| 20180009 | 2 | T | G |
| 20180010 | 2 | T | G |
| 20180011 | 2 | T | G |
| 20180012 | 2 | CT | AG |
| 20180013 | 2 | CT | AG |
| 20180014 | 2 | CT | AG |
| 20180015 | 2 | T | G |
| 20180016 | 2 | CT | AG |
| 20180017 | 2 | CT | AG |
| 20180018 | 2 | T | G |
| 20180019 | 2 | T | G |
| 20180020 | 2 | CT | AG |
| 20180021 | 2 | T | G |
| 20180022 | 2 | CT | AG |
| 20180023 | 2 | T | G |
| 20180024 | 2 | CT | AG |
| 20180025 | 2 | C | A |
| 20180026 | 2 | CT | AG |
| 20180027 | 2 | T | G |
| 20180028 | 2 | T | G |
| 20180029 | 2 | CT | AG |
| 20180031 | 2 | CT | AG |
| 20180032 | 2 | T | G |
| 20180033 | 2 | CT | AG |
| 20180034 | 2 | T | G |
| 20180035 | 2 | T | G |
| 20180036 | 2 | T | G |
| 20180037 | 2 | T | G |
| 20180038 | 2 | CT | AG |
| 20180039 | 2 | CT | AG |
| 20180040 | 2 | CT | AG |
| 20180041 | 2 | CT | AG |
| 20180042 | 2 | T | G |
| 20180043 | 2 | CT | AG |
| 20180044 | 2 | C | A |
| 20180045 | 2 | C | AG |
| 20180046 | 2 | T | G |
| 20180047 | 2 | CT | AG |
| 20180048 | 2 | T | G |
| 20180049 | 2 | CT | AG |
| 20180050 | 2 | C | AG |
| 20180051 | 2 | C | A |
| 20180053 | 2 | T | G |
| 20180054 | 2 | CT | G |
| 20180055 | 2 | T | G |
| 20180056 | 2 | CT | AG |
| 20180057 | 2 | CT | AG |
| 20180058 | 2 | T | G |
| 20180059 | 2 | T | G |
| 20180060 | 2 | CT | AG |
| 20180061 | 2 | T | G |
| 20180062 | 2 | T | G |
| 20180063 | 2 | T | G |
| 20180064 | 2 | T | G |
| 20180065 | 2 | T | G |
| 20180066 | 2 | T | G |
| 20180067 | 2 | CT | AG |
| 20180068 | 2 | C | AG |
| 20180069 | 2 | CT | AG |
| 20180070 | 2 | C | AG |
| 20180071 | 2 | C | N |
| 20180072 | 2 | C | A |
| 20180073 | 2 | CT | AG |
| 20180074 | 2 | T | G |
| 20180075 | 2 | N | N |
| 20180076 | 2 | T | G |
| 20180077 | 2 | T | G |
| 20180078 | 2 | CT | AG |
| 20180079 | 2 | CT | AG |
| 20180080 | 2 | T | G |
| 20180081 | 2 | T | G |
| 20180082 | 2 | T | G |
| 20180083 | 2 | CT | AG |
| 20180084 | 2 | CT | AG |
| 20180085 | 2 | C | G |
| 20180086 | 2 | CT | AG |
| 20180087 | 2 | CT | AG |
| 20180088 | 2 | C | AG |
| 20180092 | 2 | CT | AG |
| 20180093 | 2 | CT | AG |
| 20180094 | 2 | T | G |
| 20180095 | 2 | CT | AG |
| 20180096 | 2 | C | A |
| 20180097 | 2 | C | A |
| 20180098 | 2 | CT | AG |
| 20180099 | 2 | C | AG |
| 20180100 | 2 | T | G |
| 20180101 | 2 | T | G |
| 20180102 | 2 | C | A |
| 20180103 | 2 | CT | AG |
| 20180104 | 2 | T | G |
| 20180105 | 2 | C | A |
| 20180106 | 2 | CT | N |
| 20180107 | 2 | T | G |
| 20180108 | 2 | C | A |
| 20180109 | 2 | CT | G |
| 20180110 | 2 | CT | G |
| 20180111 | 2 | T | G |
| 20180112 | 2 | CT | AG |
| 20180113 | 2 | C | A |
| 20180114 | 2 | CT | AG |
| 20180115 | 2 | CT | AG |
| 20180116 | 2 | C | A |
| 20180118 | 2 | T | G |
| 20180119 | 2 | CT | AG |
| 20180120 | 2 | CT | AG |
| 20180121 | 2 | CT | AG |
| 20180122 | 2 | T | G |
| 20180124 | 2 | T | G |
| 20180125 | 2 | CT | AG |
| 20180126 | 2 | T | G |
| 20180127 | 2 | T | G |
| 20180128 | 2 | T | G |
| 20180129 | 2 | CT | AG |
| 20180130 | 2 | CT | AG |
| 20180131 | 2 | CT | AG |
| 20180132 | 2 | C | AG |
| 20180133 | 2 | T | G |
| 20180134 | 2 | CT | AG |
| 20180135 | 2 | T | G |
| 20180136 | 2 | T | G |
| 20180137 | 2 | T | G |
| 20180138 | 2 | T | G |
| 20180139 | 2 | CT | AG |
| 20180140 | 2 | CT | AG |
| 20180141 | 2 | T | G |
| 20180142 | 2 | T | G |
| 20180143 | 2 | CT | AG |
| 20180144 | 2 | T | G |
| 20180145 | 2 | T | G |
| 20180146 | 2 | CT | AG |
| 20180147 | 2 | CT | AG |
| 20180148 | 2 | T | G |
| 20180149 | 2 | T | G |
| 20180150 | 2 | CT | AG |
| 20180151 | 2 | T | G |
| 20180152 | 2 | CT | AG |
| 20180153 | 2 | T | G |
| 20180154 | 2 | CT | AG |
| 20180155 | 2 | CT | AG |
| 20180156 | 2 | T | G |
| 20180157 | 2 | CT | AG |
| 20180158 | 2 | C | A |
| 20180159 | 2 | CT | AG |
| 20180160 | 2 | T | G |
| 20180161 | 2 | T | G |
| 20180162 | 2 | T | G |
| 20180163 | 2 | CT | AG |
| 20180164 | 2 | T | G |
| 20180165 | 2 | CT | AG |
| 20180166 | 2 | T | G |
| 20180167 | 2 | T | G |
| 20180168 | 2 | T | G |
| 20180169 | 2 | T | G |
| 20180170 | 2 | T | G |
| 20180171 | 2 | C | A |
| 20180173 | 2 | T | G |
| 20180174 | 2 | T | G |
| 20180175 | 2 | CT | AG |
| 20180176 | 2 | T | G |
| 20180177 | 2 | CT | AG |
| 20180178 | 2 | CT | AG |
| 20180179 | 2 | C | G |
| 20180180 | 2 | CT | AG |
| 20180181 | 2 | CT | AG |
| 20180182 | 2 | T | G |
| 20180183 | 2 | T | G |
| 20180184 | 2 | T | G |
| 20180185 | 2 | CT | G |
| 20180186 | 2 | C | A |
| 20180187 | 2 | CT | G |
| 20180188 | 2 | C | A |
| 20180189 | 2 | T | G |
| 20180190 | 2 | C | A |
| 20180191 | 2 | T | G |
| 20180193 | 2 | T | G |
| 20180194 | 2 | CT | AG |
| 20180195 | 2 | CT | AG |
| 20180196 | 2 | C | A |
| 20180197 | 2 | CT | AG |
| 20180198 | 2 | C | A |
| 20180199 | 2 | CT | AG |
| 20180200 | 2 | T | G |
| 20180201 | 2 | T | G |
| 20180202 | 2 | T | G |
| 20180203 | 2 | CT | AG |
| 20180205 | 2 | CT | AG |
| 20180206 | 2 | T | G |
| 20180207 | 2 | T | G |
| 20180208 | 2 | T | G |
| 20180209 | 2 | T | G |
| 20180210 | 2 | CT | AG |
| 20180211 | 2 | T | G |
| 20180212 | 2 | CT | AG |
| 20180213 | 2 | T | G |
| 20180214 | 2 | T | G |
| 20180215 | 2 | T | G |
| 20180216 | 2 | T | G |
| 20180217 | 2 | CT | AG |
| 20180218 | 2 | T | G |
| 20180219 | 2 | T | G |
| 20180220 | 2 | CT | AG |
| 20180221 | 2 | T | G |
| 20180222 | 2 | CT | AG |
| 20180223 | 2 | CT | AG |
| 20180224 | 2 | T | G |
| 20180225 | 2 | CT | G |
| 20180226 | 2 | CT | G |
| 20180227 | 2 | CT | AG |
| 20180228 | 2 | C | A |
| 20180229 | 2 | CT | AG |
| 20180230 | 2 | T | G |
| 20180231 | 2 | CT | G |
| 20180232 | 2 | T | G |
| 20180233 | 2 | C | A |
| 20180234 | 2 | T | G |
| 20180235 | 2 | T | G |
| 20180236 | 2 | CT | AG |
| 20180238 | 2 | T | G |
| 20180241 | 2 | T | G |
| 20180242 | 2 | CT | AG |
| 20180243 | 2 | CT | AG |
| 20180244 | 2 | C | A |
| 20180245 | 2 | T | G |
| 20180246 | 2 | T | G |
| 20180247 | 2 | CT | AG |
| 20180248 | 2 | T | G |
| 20180249 | 2 | CT | AG |
| 20180250 | 2 | T | G |
| 20180251 | 2 | T | G |
| 20180252 | 2 | T | G |
| 20180253 | 2 | T | G |
| 20180254 | 2 | CT | AG |
| 20180255 | 2 | T | G |
| 20180256 | 2 | T | G |
| 20180257 | 2 | CT | AG |
| 20180258 | 2 | CT | AG |
| 20180259 | 2 | CT | AG |
| 20180260 | 2 | CT | AG |
| 20180261 | 2 | C | A |
| 20180262 | 2 | T | G |
| 20180263 | 2 | CT | G |
| 20180264 | 2 | CT | AG |
| 20180265 | 2 | T | G |
| 20180266 | 2 | T | G |
| 20180267 | 2 | CT | AG |
| 20180269 | 2 | CT | AG |
| 20180271 | 2 | T | G |
| 20180272 | 2 | CT | AG |
| 20180273 | 2 | T | G |
| 20180274 | 2 | T | G |
| 20180275 | 2 | T | G |
| 20180276 | 2 | CT | AG |
| 20180277 | 2 | CT | AG |
| 20180278 | 2 | T | G |
| 20180279 | 2 | T | G |
| 20180280 | 2 | CT | G |
| 20180281 | 2 | T | G |
| 20180282 | 2 | T | G |
| 20180283 | 2 | T | G |
| 20180284 | 2 | CT | G |
| 20180285 | 2 | T | G |
| 20180286 | 2 | C | AG |
| 20180287 | 2 | T | G |
| 20180288 | 2 | CT | AG |
| 20180289 | 2 | C | A |
| 20180290 | 2 | CT | G |
| 20180291 | 2 | T | G |
| 20180292 | 2 | CT | AG |
| 20180293 | 2 | T | G |
| 20180294 | 2 | CT | G |
| 20180295 | 2 | T | G |
| 20180296 | 2 | CT | AG |
| 20180297 | 2 | CT | AG |
| 20180298 | 2 | T | G |
| 20180299 | 2 | CT | AG |
| 20180300 | 2 | CT | AG |
| 20180301 | 2 | C | A |
| 20180302 | 2 | T | G |
| 20180303 | 2 | T | G |
| 20180304 | 2 | T | G |
| 20180305 | 2 | CT | G |
| 20180306 | 2 | CT | AG |
| 20180307 | 2 | CT | AG |
| 20180308 | 2 | T | G |
| 20180309 | 2 | T | G |
| 20180310 | 2 | CT | AG |
| 20180311 | 2 | T | G |
| 20180312 | 2 | CT | G |
| 20180313 | 2 | T | G |
| 20180314 | 2 | T | G |
| 20180315 | 2 | T | G |
| 20180316 | 2 | CT | G |
| 20180317 | 2 | C | A |
| 20180318 | 2 | CT | AG |
| 20180320 | 2 | T | G |
| 20180321 | 2 | C | A |
| 20180322 | 2 | T | G |
| 20180323 | 2 | T | G |
| 20180324 | 2 | T | G |
| 20180327 | 2 | T | G |
| 20180328 | 2 | C | A |
| 20180329 | 2 | CT | AG |
| 20180330 | 2 | CT | G |
| 20180331 | 2 | CT | AG |
| 20180332 | 2 | T | G |
| 20180333 | 2 | T | G |
| 20180334 | 2 | CT | G |
| 20180335 | 2 | CT | AG |
| 20180336 | 2 | C | A |
| 20180337 | 2 | T | G |
| 20180338 | 2 | C | A |
| 20180339 | 2 | CT | AG |
| 20180340 | 2 | T | G |
| 20180341 | 2 | T | G |
| 20180342 | 2 | T | G |
| 20180343 | 2 | T | G |
| 20180344 | 2 | CT | AG |
| 20180345 | 2 | CT | AG |
| 20180346 | 2 | T | G |
| 20180347 | 2 | C | A |
| 20180348 | 2 | CT | G |
| 20180349 | 2 | T | G |
| 20180350 | 2 | CT | G |
| 20180351 | 2 | C | A |
| 20180352 | 2 | CT | AG |
| 20180353 | 2 | CT | AG |
| 20180354 | 2 | CT | AG |
| 20180355 | 2 | C | A |
| 20180356 | 2 | CT | AG |
| 20180359 | 2 | CT | AG |
| 20180360 | 2 | C | A |
| 20180361 | 2 | T | G |
| 20180362 | 2 | T | G |
| 20180363 | 2 | C | AG |
| 20180364 | 2 | T | G |
| 20180365 | 2 | CT | AG |
| 20180366 | 2 | T | G |
| 20180367 | 2 | C | A |
| 20180368 | 2 | T | G |
| 20180369 | 2 | CT | AG |
| 20180370 | 2 | CT | AG |
| 20180371 | 2 | T | G |
| 20180372 | 2 | T | G |
| 20180373 | 2 | CT | AG |
| 20180374 | 2 | T | G |
| 20180375 | 2 | CT | AG |
| 20180376 | 2 | CT | AG |
| 20180377 | 2 | CT | AG |
| 20180378 | 2 | CT | AG |
| 20180379 | 2 | CT | N |
| 20180380 | 2 | CT | AG |
| 20180381 | 2 | T | G |
| 20180382 | 2 | CT | AG |
| 20180383 | 2 | C | A |
| 20180384 | 2 | C | A |
| 20180385 | 2 | CT | AG |
| 20180386 | 2 | T | G |
| 20180387 | 2 | CT | AG |
| 20180388 | 2 | CT | G |
| 20180389 | 2 | T | G |
| 20180390 | 2 | C | A |
| 20180391 | 2 | CT | AG |
| 20180392 | 2 | T | G |
| 20180393 | 2 | T | G |
| 20180394 | 2 | CT | AG |
| 20180395 | 2 | CT | AG |
| 20180396 | 2 | CT | AG |
| 20180397 | 2 | T | G |
| 20180398 | 2 | T | G |
| 20180399 | 2 | CT | AG |
| 20180400 | 2 | T | G |
| 20180401 | 2 | T | G |
| 20180402 | 2 | CT | AG |
| 20180403 | 2 | T | G |
| 20180404 | 2 | T | G |
| 20180405 | 2 | CT | AG |
| 20180406 | 2 | CT | G |
| 20180407 | 2 | T | G |
| 20180408 | 2 | CT | AG |
| 20180409 | 2 | CT | AG |
| 20180410 | 2 | CT | AG |
| 20180411 | 2 | T | G |
| 20180412 | 2 | T | G |
| 20180413 | 2 | CT | AG |
| 20180414 | 2 | CT | AG |
| 20180415 | 2 | CT | G |
| 20180416 | 2 | CT | AG |
| 20180417 | 2 | T | G |
| 20180418 | 2 | T | G |
| 20180419 | 2 | CT | AG |
| 20180420 | 2 | T | G |
| 20180421 | 2 | CT | AG |
| 20180422 | 2 | CT | AG |
| 20180423 | 2 | CT | G |
| 20180424 | 2 | CT | AG |
| 20180425 | 2 | CT | AG |
| 20180426 | 2 | CT | AG |
| 20180427 | 2 | T | G |
| 20180428 | 2 | T | G |
| 20180429 | 2 | C | A |
| 20180431 | 2 | C | A |
| 20180432 | 2 | CT | AG |
| 20180433 | 2 | T | G |
| 20180434 | 2 | C | AG |
| 20180436 | 2 | T | G |
| 20180437 | 2 | T | G |
| 20180438 | 2 | CT | AG |
| 20180439 | 2 | CT | AG |
| 20180440 | 2 | CT | AG |
| 20180441 | 2 | T | G |
| 20180442 | 2 | T | G |
| 20180443 | 2 | CT | AG |
| 20180444 | 2 | C | A |
| 20180445 | 2 | T | G |
| 20180446 | 2 | CT | AG |
| 20180447 | 2 | CT | AG |
| 20180449 | 2 | T | G |
| 20180450 | 2 | CT | AG |
| 20180451 | 2 | CT | G |
| 20180452 | 2 | C | A |
| 20180453 | 2 | C | A |
| 20180454 | 2 | T | G |
| 20180455 | 2 | CT | AG |
| 20180456 | 2 | CT | AG |
| 20180457 | 2 | CT | AG |
| 20180458 | 2 | CT | AG |
| 20180459 | 2 | CT | AG |
| 20180460 | 2 | T | G |
| 20180461 | 2 | CT | AG |
| 20180462 | 2 | CT | AG |
| 20180463 | 2 | CT | AG |
| 20180464 | 2 | T | G |
| 20180465 | 2 | CT | AG |
| 20180466 | 2 | T | G |
| 20180467 | 2 | CT | AG |
| 20180468 | 2 | T | G |
| 20180469 | 2 | CT | AG |
| 20180470 | 2 | CT | AG |
| 20180472 | 2 | CT | AG |
| 20180473 | 2 | CT | N |
| 20180474 | 2 | T | G |
| 20180475 | 2 | T | G |
| 20180476 | 2 | C | A |
| 20180477 | 2 | C | A |
| 20180478 | 2 | CT | AG |
| 20180479 | 2 | C | A |
| 20180480 | 2 | T | G |
| 20180481 | 2 | T | N |
| 20180482 | 2 | C | A |
| 20180483 | 2 | C | A |
| 20180484 | 2 | C | AG |
| 20180485 | 2 | T | G |
| 20180486 | 2 | T | G |
| 20180487 | 2 | T | G |
| 20180488 | 2 | T | G |
| 20180489 | 2 | C | N |
| 20180490 | 2 | CT | AG |
| 20180491 | 2 | T | G |
| 20180492 | 2 | T | G |
| 20180493 | 2 | T | G |
| 20180494 | 2 | CT | AG |
| 20180495 | 2 | C | A |
| 20180496 | 2 | C | A |
| 20180497 | 2 | CT | AG |
| 20180498 | 2 | T | G |
| 20180499 | 2 | CT | AG |
| 20180500 | 2 | CT | AG |
| 20180501 | 2 | T | G |
| 20180502 | 2 | CT | AG |
| 20180503 | 2 | CT | AG |
| 20180504 | 2 | T | G |
| 20180505 | 2 | T | G |
| 20180506 | 2 | T | G |
| 20180507 | 2 | CT | AG |
| 20180508 | 2 | CT | AG |
| 20180509 | 2 | T | G |
| 20180510 | 2 | CT | AG |
| 20180511 | 2 | CT | AG |
| 20180512 | 2 | CT | AG |
| 20180513 | 2 | T | G |
| 20180514 | 2 | C | A |
| 20180515 | 2 | CT | AG |
| 20180516 | 2 | CT | AG |
| 20180517 | 2 | CT | AG |
| 20180518 | 2 | CT | G |
| 20180519 | 2 | T | G |
| 20180520 | 2 | T | G |
| 20180521 | 2 | T | G |
| 20180522 | 2 | T | G |
| 20180523 | 2 | C | A |
| 20180524 | 2 | CT | AG |
| 20180525 | 2 | CT | AG |
| 20180526 | 2 | T | G |
| 20180527 | 2 | CT | AG |
| 20180528 | 2 | C | AG |
| 20180529 | 2 | T | G |
| 20180530 | 2 | T | G |
| 20180531 | 2 | T | G |
| 20180532 | 2 | CT | AG |
| 20180533 | 2 | T | G |
| 20180534 | 2 | CT | AG |
| 20180535 | 2 | CT | AG |
| 20180536 | 2 | CT | AG |
| 20180537 | 2 | CT | AG |
| 20180538 | 2 | T | G |
| 20180539 | 2 | T | G |
| 20180540 | 2 | T | G |
| 20180541 | 2 | CT | AG |
| 20180542 | 2 | C | A |
| 20180543 | 2 | C | AG |
| 20180544 | 2 | T | G |
| 20180545 | 2 | T | G |
| 20180546 | 2 | CT | AG |
| 20180547 | 2 | CT | G |
| 20180548 | 2 | CT | AG |
| 20180549 | 2 | T | G |
| 20180550 | 2 | T | G |
| 20180551 | 2 | C | A |
| 20180552 | 2 | CT | AG |
| 20180553 | 2 | T | G |
| 20180554 | 2 | CT | AG |
| 20180555 | 2 | CT | AG |
| 20180556 | 2 | CT | AG |
| 20180557 | 2 | CT | AG |
| 20180558 | 2 | T | G |
| 20180559 | 2 | T | G |
| 20180560 | 2 | CT | AG |
| 20180561 | 2 | CT | AG |
| 20180562 | 2 | C | A |
| 20180563 | 2 | CT | AG |
| 20180564 | 2 | T | G |
| 20180565 | 2 | C | AG |
| 20180566 | 2 | CT | G |
| 20180567 | 2 | CT | AG |
| 20180568 | 2 | T | G |
| 20180569 | 2 | CT | AG |
| 20180570 | 2 | T | G |
| 20180571 | 2 | T | G |
| 20180572 | 2 | CT | AG |
| 20180573 | 2 | CT | AG |
| 20180574 | 2 | C | A |
| 20180575 | 2 | CT | AG |
| 20180576 | 2 | CT | AG |
| 20180577 | 2 | CT | AG |
| 20180578 | 2 | CT | AG |
| 20180579 | 2 | C | A |
| 20180580 | 2 | CT | AG |
| 20180581 | 2 | CT | AG |
| 20180582 | 2 | C | A |
| 20180583 | 2 | CT | AG |
| 20180584 | 2 | CT | AG |
| 20180585 | 2 | CT | AG |
| 20180586 | 2 | C | A |
| 20180587 | 2 | CT | AG |
| 20180588 | 2 | T | G |
| 20180589 | 2 | T | G |
| 20180590 | 2 | T | G |
| 20180591 | 2 | CT | G |
| 20180592 | 2 | T | G |
| 20180593 | 2 | CT | AG |
| 20180594 | 2 | C | A |
| 20180595 | 2 | T | G |
| 20180596 | 2 | T | G |
| 20180597 | 2 | CT | G |
| 20180598 | 2 | CT | AG |
| 20180599 | 2 | CT | AG |
| 20180600 | 2 | CT | AG |
| 20180601 | 2 | C | AG |
| 20180602 | 2 | CT | AG |
| 20180603 | 2 | CT | AG |
| 20180604 | 2 | T | G |
| 20180605 | 2 | T | G |
| 20180606 | 2 | T | G |
| 20180607 | 2 | C | AG |
| 20180608 | 2 | C | A |
| 20180609 | 2 | T | G |
| 20180610 | 2 | CT | AG |
| 20180611 | 2 | CT | AG |
| 20180612 | 2 | C | G |
| 20180613 | 2 | CT | AG |
| 20180614 | 2 | T | G |
| 20180615 | 2 | T | G |
| 20180616 | 2 | T | G |
| 20180617 | 2 | T | G |
| 20180618 | 2 | T | G |
| 20180619 | 2 | CT | G |
| 20180620 | 2 | CT | G |
| 20180621 | 2 | T | G |
| 20180622 | 2 | T | G |
| 20180623 | 2 | CT | AG |
| 20180624 | 2 | CT | AG |
| 20180625 | 2 | T | G |
| 20180626 | 2 | T | G |
| 20180627 | 2 | T | G |
| 20180628 | 2 | C | AG |
| 20180629 | 2 | T | G |
| 20180630 | 2 | T | G |
| 20180631 | 2 | CT | AG |
| 20180632 | 2 | CT | AG |
| 20180633 | 2 | CT | AG |
| 20180634 | 2 | T | G |
| 20180635 | 2 | T | G |
| 20180636 | 2 | T | G |
| 20180637 | 2 | T | G |
| 20180638 | 2 | T | G |
| 20180639 | 2 | T | G |
| 20180640 | 2 | T | G |
| 20180641 | 2 | T | G |
| 20180643 | 2 | T | G |
| 20180644 | 2 | C | AG |
| 20180645 | 2 | T | G |
| 20180646 | 2 | T | G |
| 20180647 | 2 | C | A |
| 20180648 | 2 | CT | AG |
| 20180649 | 2 | CT | AG |
| 20180650 | 2 | CT | AG |
| 20180651 | 2 | T | G |
| 20180652 | 2 | T | G |
| 20180653 | 2 | CT | AG |
| 20180654 | 2 | C | A |
| 20180655 | 2 | T | G |
| 20180656 | 2 | T | G |
| 20180657 | 2 | CT | AG |
| 20180658 | 2 | CT | G |
| 20180659 | 2 | C | A |
| 20180660 | 2 | T | G |
| 20180661 | 2 | T | G |
| 20180662 | 2 | T | G |
| 20180663 | 2 | CT | AG |
| 20180664 | 2 | T | G |
| 20180665 | 2 | T | G |
| 20180666 | 2 | CT | AG |
| 20180667 | 2 | T | G |
| 20180668 | 2 | CT | AG |
| 20180669 | 2 | T | G |
| 20180670 | 2 | CT | AG |
| 20180671 | 2 | CT | AG |
| 20180672 | 2 | CT | AG |
| 20180673 | 2 | CT | AG |
| 20180674 | 2 | T | G |
| 20180675 | 2 | T | G |
| 20180676 | 2 | CT | AG |
| 20180677 | 2 | T | G |
| 20180678 | 2 | CT | AG |
| 20180679 | 2 | CT | AG |
| 20180680 | 2 | CT | AG |
| 20180681 | 2 | CT | AG |
| 20180682 | 2 | T | G |
| 20180683 | 2 | CT | AG |
| 20180684 | 2 | T | G |
| 20180685 | 2 | CT | AG |
| 20180686 | 2 | T | G |
| 20180687 | 2 | T | G |
| 20180688 | 2 | T | G |
| 20180689 | 2 | T | G |
| 20180690 | 2 | CT | AG |
| 20180691 | 2 | C | A |
| 20180692 | 2 | T | G |
| 20180693 | 2 | CT | AG |
| 20180694 | 2 | CT | AG |
| 20180695 | 2 | T | G |
| 20180696 | 2 | CT | AG |
| 20180697 | 2 | T | G |
| 20180698 | 2 | T | G |
| 20180699 | 2 | C | A |
| 20180700 | 2 | T | G |
| 20180701 | 2 | CT | AG |
| 20180702 | 2 | T | G |
| 20180703 | 2 | CT | AG |
| 20180704 | 2 | T | G |
| 20180705 | 2 | T | G |
| 20180706 | 2 | C | A |
| 20180707 | 2 | C | A |
| 20180708 | 2 | C | A |
| 20180709 | 2 | CT | G |
| 20180710 | 2 | CT | AG |
| 20180711 | 2 | CT | AG |
| 20180712 | 2 | C | A |
| 20180713 | 2 | CT | AG |
| 20180714 | 2 | CT | AG |
| 20180715 | 2 | T | G |
| 20180716 | 2 | T | G |
| 20180717 | 2 | CT | AG |
| 20180718 | 2 | C | A |
| 20180719 | 2 | T | G |
| 20180720 | 2 | T | G |
| 20180721 | 2 | CT | AG |
| 20180722 | 2 | T | G |
| 20180723 | 2 | CT | AG |
| 20180724 | 2 | CT | AG |
| 20180725 | 2 | CT | G |
| 20180726 | 2 | T | G |
| 20180727 | 2 | T | G |
| 20180728 | 2 | T | G |
| 20180729 | 2 | T | G |
| 20180730 | 2 | CT | AG |
| 20180731 | 2 | CT | AG |
| 20180732 | 2 | C | A |
| 20180734 | 2 | CT | AG |
| 20180735 | 2 | T | G |
| 20180736 | 2 | CT | AG |
| 20180737 | 2 | C | G |
| 20180738 | 2 | CT | G |
| 20180739 | 2 | CT | AG |
| 20180740 | 2 | CT | G |
| 20180741 | 2 | T | G |
| 20180742 | 2 | T | G |
| 20180743 | 2 | T | G |
| 20180744 | 2 | CT | G |
| 20180745 | 2 | CT | AG |
| 20180746 | 2 | T | G |
| 20180747 | 2 | C | A |
| 20180748 | 2 | CT | AG |
| 20180749 | 2 | CT | G |
| 20180750 | 2 | T | G |
| 20180751 | 2 | T | G |
| 20180752 | 2 | CT | AG |
| 20180753 | 2 | CT | G |
| 20180754 | 2 | CT | AG |
| 20180755 | 2 | T | G |
| 20180756 | 2 | T | G |
| 20180757 | 2 | T | G |
| 20180758 | 2 | T | G |
| 20180759 | 2 | T | G |
| 20180760 | 2 | T | G |
| 20180761 | 2 | T | G |
| 20180762 | 2 | T | G |
| 20180763 | 2 | CT | AG |
| 20180764 | 2 | CT | AG |
| 20180765 | 2 | CT | AG |
| 20180766 | 2 | T | G |
| 20180767 | 2 | T | G |
| 20180768 | 2 | T | G |
| 20180769 | 2 | CT | AG |
| 20180770 | 2 | T | G |
| 20180771 | 2 | T | G |
| 20180772 | 2 | T | G |
| 20180773 | 2 | T | G |
| 20180774 | 2 | CT | AG |
| 20180775 | 2 | T | G |
| 20180776 | 2 | T | G |
| 20180777 | 2 | CT | G |
| 20180778 | 2 | T | G |
| 20180779 | 2 | CT | AG |
| 20180780 | 2 | CT | AG |
| 20180781 | 2 | T | G |
| 20180782 | 2 | T | G |
| 20180783 | 2 | C | A |
| 20180784 | 2 | T | G |
| 20180785 | 2 | T | G |
| 20180786 | 2 | C | A |
| 20180787 | 2 | CT | AG |
| 20180788 | 2 | CT | AG |
| 20180789 | 2 | CT | AG |
| 20180790 | 2 | CT | G |
| 20180791 | 2 | T | G |
| 20180792 | 2 | CT | AG |
| 20180793 | 2 | C | A |
| 20180794 | 2 | T | G |
| 20180795 | 2 | CT | AG |
| 20180796 | 2 | T | G |
| 20180797 | 2 | CT | AG |
| 20180798 | 2 | T | G |
| 20180799 | 2 | CT | AG |
| 20180800 | 2 | T | G |
| 20180801 | 2 | CT | AG |
| 20180802 | 2 | C | A |
| 20180803 | 2 | C | A |
| 20180804 | 2 | C | A |
| 20180805 | 2 | CT | G |
| 20180806 | 2 | T | G |
| 20180807 | 2 | T | G |
| 20180808 | 2 | T | G |
| 20180809 | 2 | CT | AG |
| 20180810 | 2 | C | AG |
| 20180811 | 2 | C | AG |
| 20180812 | 2 | CT | AG |
| 20180813 | 2 | CT | AG |
| 20180814 | 2 | T | G |
| 20180815 | 2 | T | G |
| 20180816 | 2 | CT | AG |
| 20180817 | 2 | C | A |
| 20180818 | 2 | T | G |
| 20180819 | 2 | T | G |
| 20180820 | 2 | CT | AG |
| 20180821 | 2 | T | G |
| 20180822 | 2 | C | A |
| 20180823 | 2 | CT | AG |
| 20180824 | 2 | CT | AG |
| 20180825 | 2 | T | G |
| 20180826 | 2 | T | G |
| 20180827 | 2 | T | G |
| 20180828 | 2 | CT | G |
| 20180829 | 2 | CT | AG |
| 20180830 | 2 | T | G |
| 20180831 | 2 | T | G |
| 20180832 | 2 | CT | AG |
| 20180833 | 2 | CT | G |
| 20180834 | 2 | CT | G |
| 20180835 | 2 | CT | AG |
| 20180836 | 2 | T | G |
| 20180837 | 2 | CT | AG |
| 20180838 | 2 | CT | AG |
| 20180839 | 2 | CT | AG |
| 20180840 | 2 | C | A |
| 20180841 | 2 | CT | G |
| 20180842 | 2 | CT | G |
| 20180843 | 2 | T | G |
| 20180844 | 2 | T | G |
| 20180846 | 2 | CT | AG |
| 20180847 | 2 | T | G |
| 20180848 | 2 | T | G |
| 20180849 | 2 | CT | AG |
| 20180850 | 2 | CT | AG |
| 20180851 | 2 | CT | AG |
| 20180852 | 2 | CT | G |
| 20180853 | 2 | T | G |
| 20180854 | 2 | T | G |
| 20180855 | 2 | T | G |
| 20180856 | 2 | CT | AG |
| 20180857 | 2 | T | G |
| 20180858 | 2 | T | G |
| 20180859 | 2 | T | G |
| 20180860 | 2 | T | G |
| 20180861 | 2 | T | G |
| 20180862 | 2 | CT | AG |
| 20180863 | 2 | T | G |
| 20180864 | 2 | T | G |
| 20180865 | 2 | C | A |
| 20180866 | 2 | CT | AG |
| 20180867 | 2 | T | G |
| 20180868 | 2 | C | A |
| 20180869 | 2 | CT | AG |
| 20180870 | 2 | C | AG |
| 20180871 | 2 | CT | AG |
| 20180872 | 2 | CT | AG |
| 20180873 | 2 | T | G |
| 20180874 | 2 | CT | AG |
| 20180875 | 2 | T | G |
| 20180876 | 2 | CT | AG |
| 20180877 | 2 | T | G |
| 20180878 | 2 | C | A |
| 20180879 | 2 | CT | AG |
| 20180880 | 2 | T | G |
| 20180881 | 2 | T | G |
| 20180882 | 2 | T | G |
| 20180883 | 2 | CT | AG |
| 20180884 | 2 | T | G |
| 20180885 | 2 | CT | AG |
| 20180886 | 2 | CT | AG |
| 20180887 | 2 | C | A |
| 20180888 | 2 | T | G |
| 20180889 | 2 | T | G |
| 20180890 | 2 | CT | AG |
| 20180891 | 2 | CT | AG |
| 20180892 | 2 | T | G |
| 20180893 | 2 | T | G |
| 20180894 | 2 | T | G |
| 20180895 | 2 | T | G |
| 20180896 | 2 | CT | AG |
| 20180897 | 2 | T | G |
| 20180898 | 2 | C | A |
| 20180899 | 2 | CT | AG |
| 20180900 | 2 | T | G |
| 20180901 | 2 | T | G |
| 20180902 | 2 | T | G |
| 20180903 | 2 | CT | AG |
| 20180904 | 2 | CT | G |
| 20180905 | 2 | CT | AG |
| 20180906 | 2 | CT | AG |
| 20180907 | 2 | CT | G |
| 20180908 | 2 | CT | AG |
| 20180909 | 2 | CT | AG |
| 20180910 | 2 | T | G |
| 20180911 | 2 | CT | AG |
| 20180912 | 2 | T | G |
| 20180913 | 2 | CT | AG |
| 20180914 | 2 | CT | AG |
| 20180915 | 2 | CT | AG |
| 20180916 | 2 | T | G |
| 20180917 | 2 | T | G |
| 20180918 | 2 | CT | AG |
| 20180919 | 2 | C | A |
| 20180920 | 2 | T | G |
| 20180921 | 2 | T | G |
| 20180922 | 2 | CT | AG |
| 20180923 | 2 | T | G |
| 20180924 | 2 | C | A |
| 20180925 | 2 | CT | AG |
| 20180926 | 2 | CT | AG |
| 20180927 | 2 | T | G |
| 20180928 | 2 | CT | AG |
| 20180930 | 2 | CT | AG |
| 20180931 | 2 | CT | A |
| 20180933 | 2 | CT | AG |
| 20180935 | 2 | CT | AG |
| 20180936 | 2 | CT | G |
| 20180937 | 2 | CT | G |
| 20180938 | 2 | C | A |
| 20180939 | 2 | CT | AG |
| 20180940 | 2 | CT | AG |
| 20180941 | 2 | CT | AG |
| 20180942 | 2 | T | G |
| 20180943 | 2 | T | G |
| 20180944 | 2 | T | G |
| 20180945 | 2 | T | G |
| 20180946 | 2 | C | A |
| 20180947 | 2 | CT | AG |
| 20180948 | 2 | CT | AG |
| 20180949 | 2 | CT | AG |
| 20180950 | 2 | CT | AG |
| 20180951 | 2 | T | G |
| 20180952 | 2 | CT | AG |
| 20180953 | 2 | T | G |
| 20180954 | 2 | T | G |
| 20180955 | 2 | T | G |
| 20180956 | 2 | T | G |
| 20180957 | 2 | T | G |
| 20180958 | 2 | CT | AG |
| 20180959 | 2 | CT | AG |
| 20180960 | 2 | T | G |
| 20180961 | 2 | C | A |
| 20180962 | 2 | CT | AG |
| 20180963 | 2 | CT | AG |
| 20180964 | 2 | CT | AG |
| 20180965 | 2 | CT | AG |
| 20180966 | 2 | C | A |
| 20180967 | 2 | CT | AG |
| 20180968 | 2 | CT | AG |
| 20180969 | 2 | T | G |
| 20180970 | 2 | T | G |
| 20180972 | 2 | CT | AG |
| 20180973 | 2 | CT | G |
| 20180975 | 2 | CT | G |
| 20180976 | 2 | T | G |
| 20180977 | 2 | CT | AG |
| 20180978 | 2 | C | AG |
| 20180979 | 2 | T | G |
| 20180981 | 2 | T | G |
| 20180982 | 2 | T | G |
| 20180983 | 2 | CT | AG |
| 20180984 | 2 | C | A |
| 20180985 | 2 | C | A |
| 20180986 | 2 | N | N |
| 20180987 | 2 | C | AG |
| 20180988 | 2 | C | A |
| 20180989 | 2 | C | A |
| 20180990 | 2 | N | N |
| 20180991 | 2 | N | N |
| 20180992 | 2 | N | N |
| 20180993 | 2 | T | G |
| 20180994 | 2 | CT | AG |
| 20180995 | 2 | T | G |
| 20180996 | 2 | CT | G |
| 20180997 | 2 | T | G |
| 20180998 | 2 | CT | AG |
| 20180999 | 2 | CT | AG |
| 20181000 | 2 | CT | AG |
| 20181001 | 2 | CT | AG |
| 20181002 | 2 | T | G |
| 20181003 | 2 | T | G |
| 20181005 | 2 | T | G |
| 20181006 | 2 | T | G |
| 20181007 | 2 | CT | AG |
| 20181008 | 2 | CT | AG |
| 20181009 | 2 | CT | G |
| 20181010 | 2 | T | G |
| 20181011 | 2 | T | G |
| 20181012 | 2 | CT | AG |
| 20181013 | 2 | CT | AG |
| 20181014 | 2 | T | G |
| 20181015 | 2 | CT | AG |
| 20181016 | 2 | T | G |
| 20181017 | 2 | C | A |
| 20181018 | 2 | T | G |
| 20181019 | 2 | CT | AG |
| 20181020 | 2 | CT | AG |
| 20181021 | 2 | T | G |
| 20181022 | 2 | T | G |
| 20181023 | 2 | C | AG |
| 20181024 | 2 | T | G |
| 20181025 | 2 | CT | AG |
| 20181026 | 2 | T | G |
| 20181027 | 2 | CT | AG |
| 20181028 | 2 | CT | AG |
| 20181029 | 2 | T | G |
| 20181030 | 2 | CT | AG |
| 20181031 | 2 | C | A |
| 20181033 | 2 | CT | AG |
| 20181034 | 2 | CT | AG |
| 20181035 | 2 | CT | AG |
| 20181036 | 2 | T | G |
| 20181037 | 2 | CT | AG |
| 20181038 | 2 | CT | AG |
| 20181040 | 2 | T | G |
| 20181041 | 2 | T | G |

Group ^a^: Label 1 stands for control group. Label 2 stands for SLE group.

rs4713853^b^: T represents Homozygous TT genotype. C represents Homozygous CC genotype. CT represents Heterozygous CT genotype.

rs2267664^c^: G represents Homozygous GG genotype. A represents Homozygous AA genotype. AG represents Heterozygous AG genotype.

Supplementary table 3. Hardy-Weinberg equilibrium for rs4713853 and rs2267664 in the replication cohort.

| SNPs | Hardy-Weinberg equilibrium ^*^ | |
| --- | --- | --- |
|  | Cases | Controls |
| rs4713853 | 0.38 | 0.291 |
| rs2267664 | 0.249 | 0.57 |

^*^: Deviations from Hardy-Weinberg equilibrium were calculated using goodness-of-fit χ^2^ test.
